# Supplementary material for: Whole-genome microsynteny-based phylogeny of angiosperms
Source: Nat Commun. 2021 Jun 9;12:3498. doi: 10.1038/s41467-021-23665-0 (PMC8190143; doi:10.1038/s41467-021-23665-0)
Supplement: Supplementary file 1 — Supplementary Information [file 41467_2021_23665_MOESM1_ESM.pdf]

Supplementary Information

Whole-genome microsynteny-based phylogeny of angiosperms

Zhao *et al*

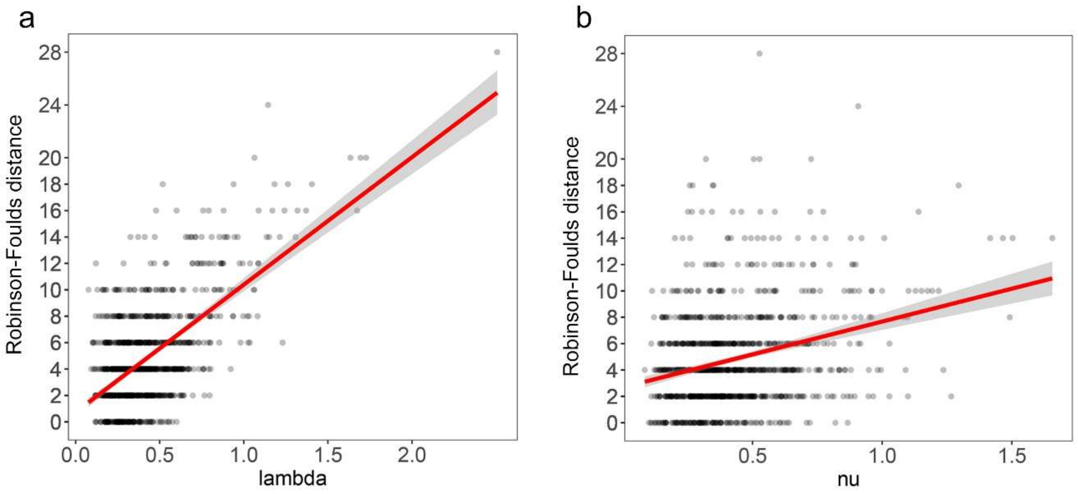

Supplementary Fig. 1 Simulations for 1000 gene families, with 1000 replicates, using the 62-plant species tree. (a) Scatterplot showing the correlation between Robinson-Foulds (RF) distance of the recovered to the true species tree and gene duplication/loss rate (λ). The grey band around the red linear regression line represents the 95% confidence interval. (b) Scatterplot showing the correlation between RF distance and gene rearrangement rate (ν). The grey band around the red linear regression line represents the 95% confidence interval.

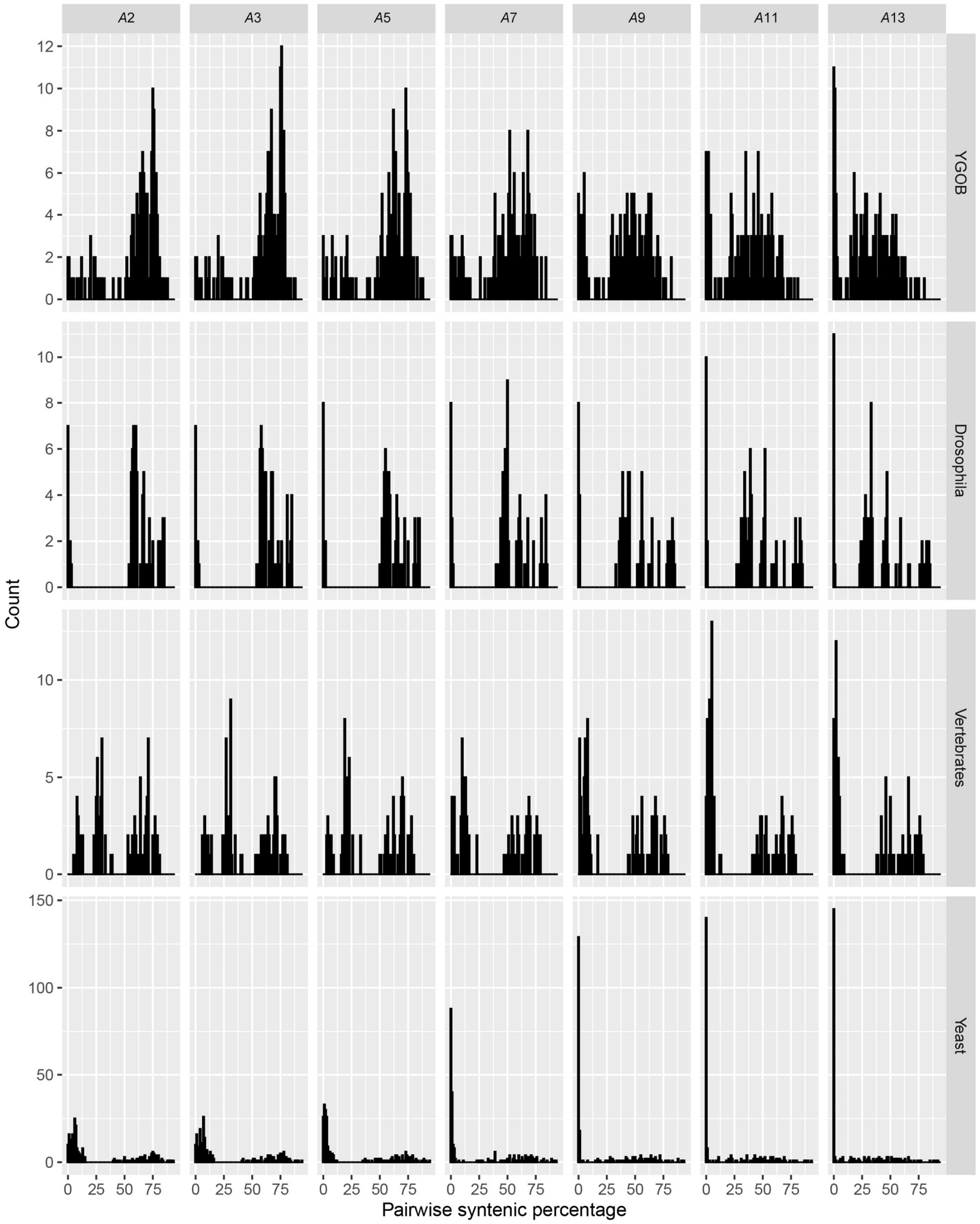

Supplementary Fig. 2 Distribution of syntenic percentages of pairwise inter- and intra-genome comparisons (which is calculated using the total number of syntenic genes relative to the total number of genes between two genomes) under different settings of the minimum number of required anchor pairs (A_{min}) for the four datasets (YGOB, *Drosophila*, vertebrates, and yeasts).

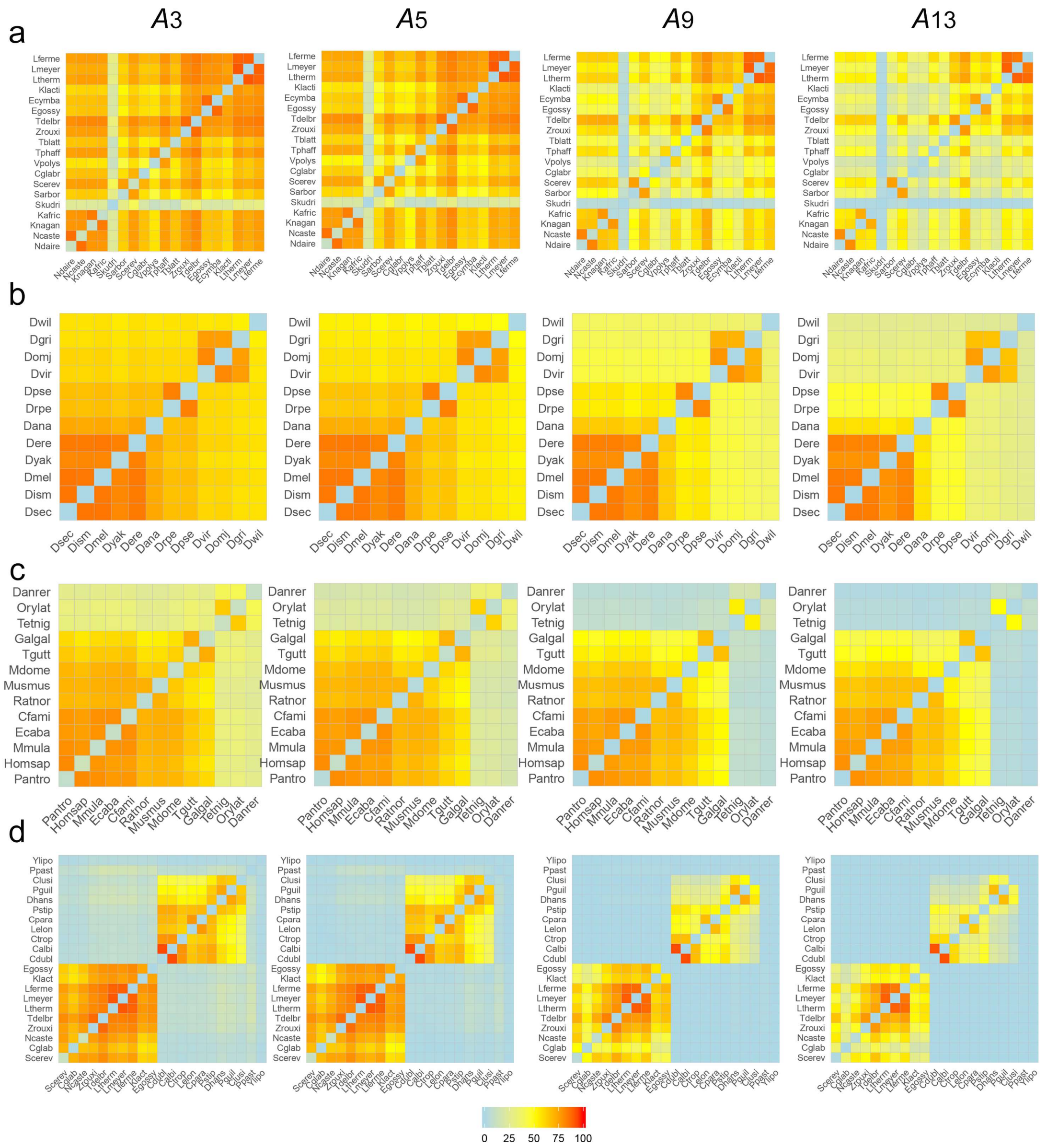

Supplementary Fig. 3 Matrices showing the changes of syntenic percentages for all pairwise genome comparisons under A_{min} settings of 3, 5, 9, and 13. The four data sets are, from top to bottom (**a**) YGOB, (**b**) *Drosophila*, (**c**) vertebrate, and (**d**) yeast. Each cell of the matrix represents an overall syntenic percentage of a genome comparison, which is calculated using the total number of syntenic genes relative to the total number of genes of the two genomes. The color indicates the values and goes from low (blue) to high (red).

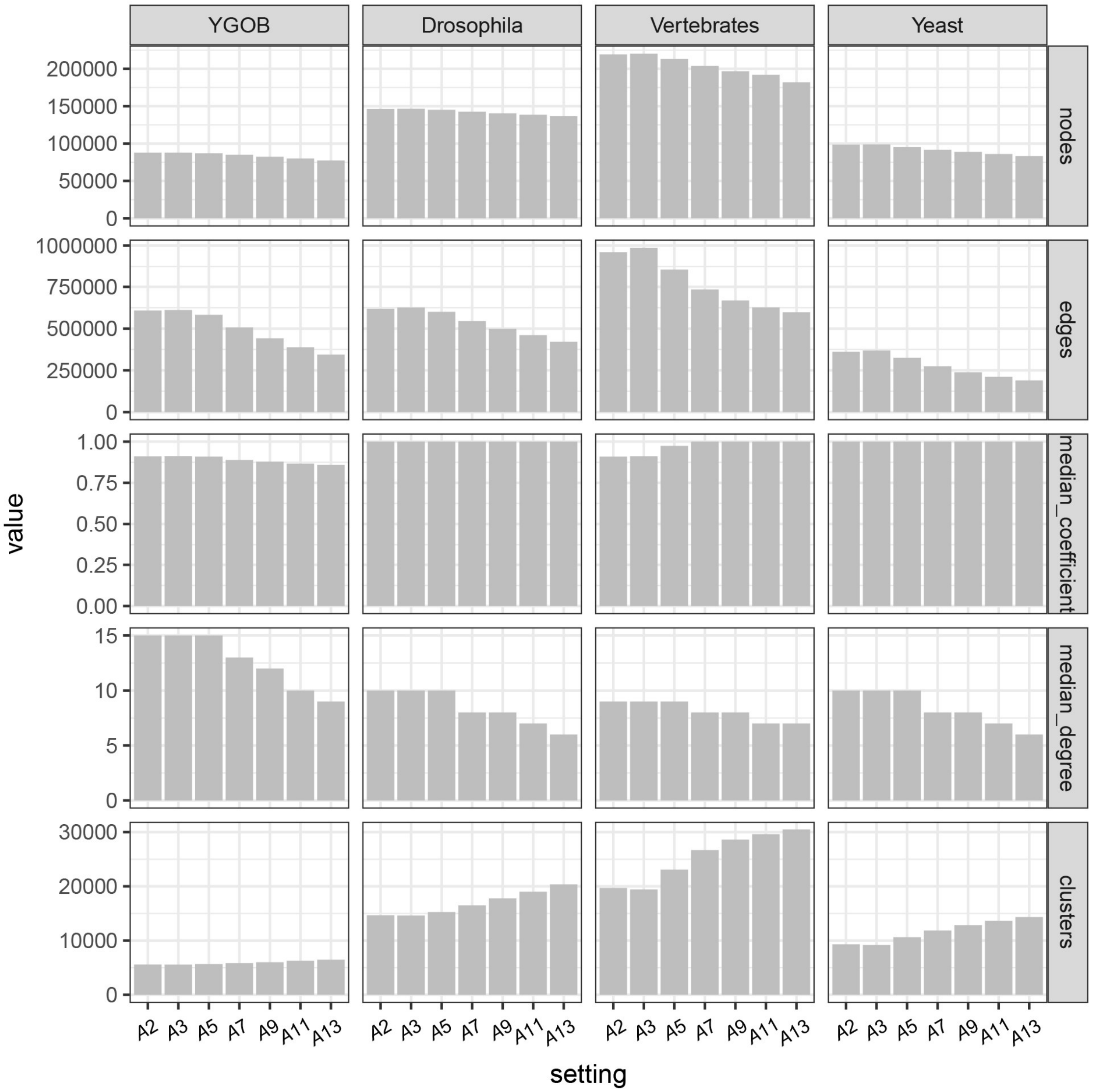

Supplementary Fig. 4 Metrics of synteny networks under different settings of A_{min}. These include numbers of nodes, edges, median clustering coefficient, median node degree, and total number of clusters. In general, stricter parameters lead to decreases in number of nodes and edges, and node degree, but create more clusters.

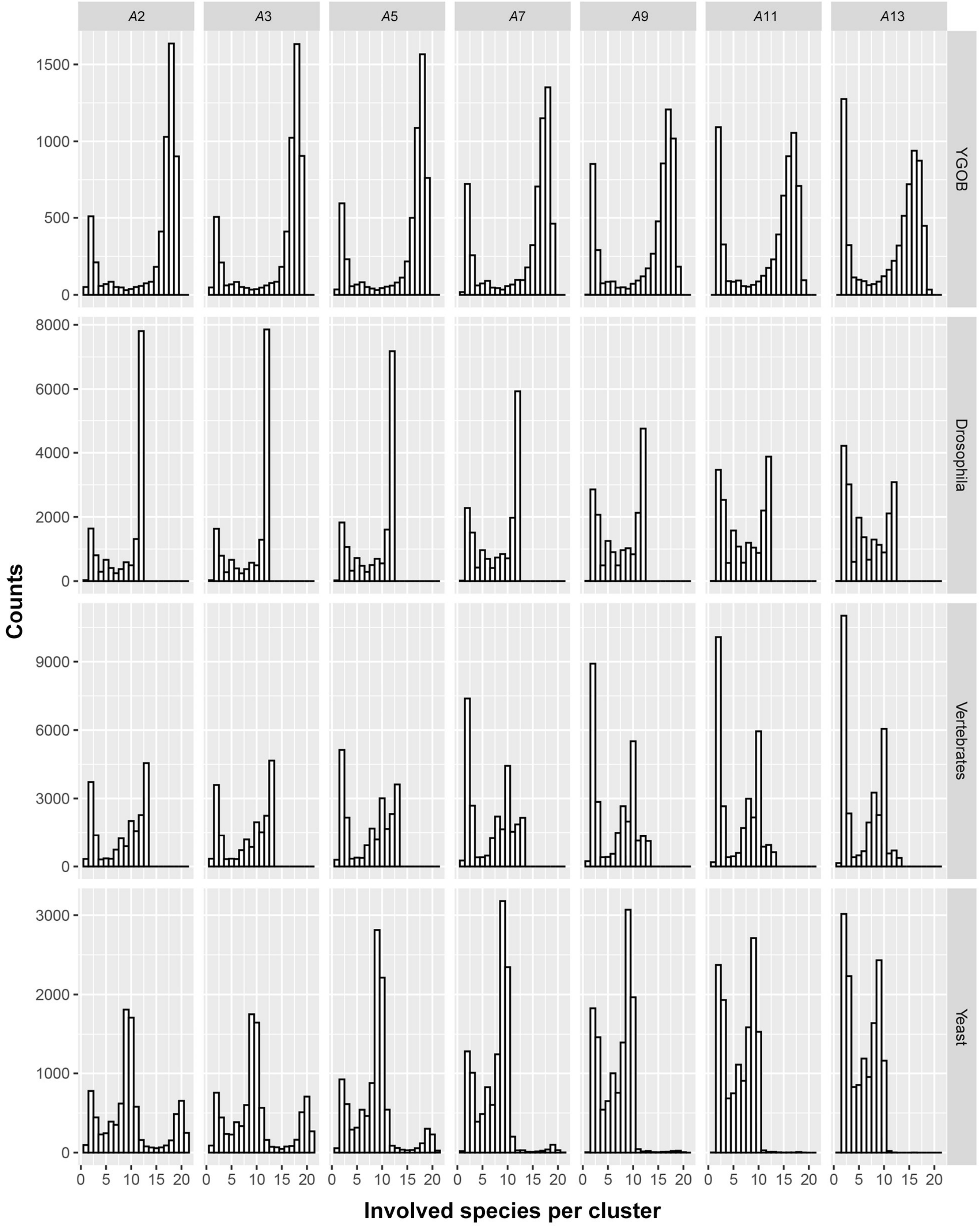

Supplementary Fig. 5 Distributions of the number of involved species in the clusters under different settings of $A_{\min}$.

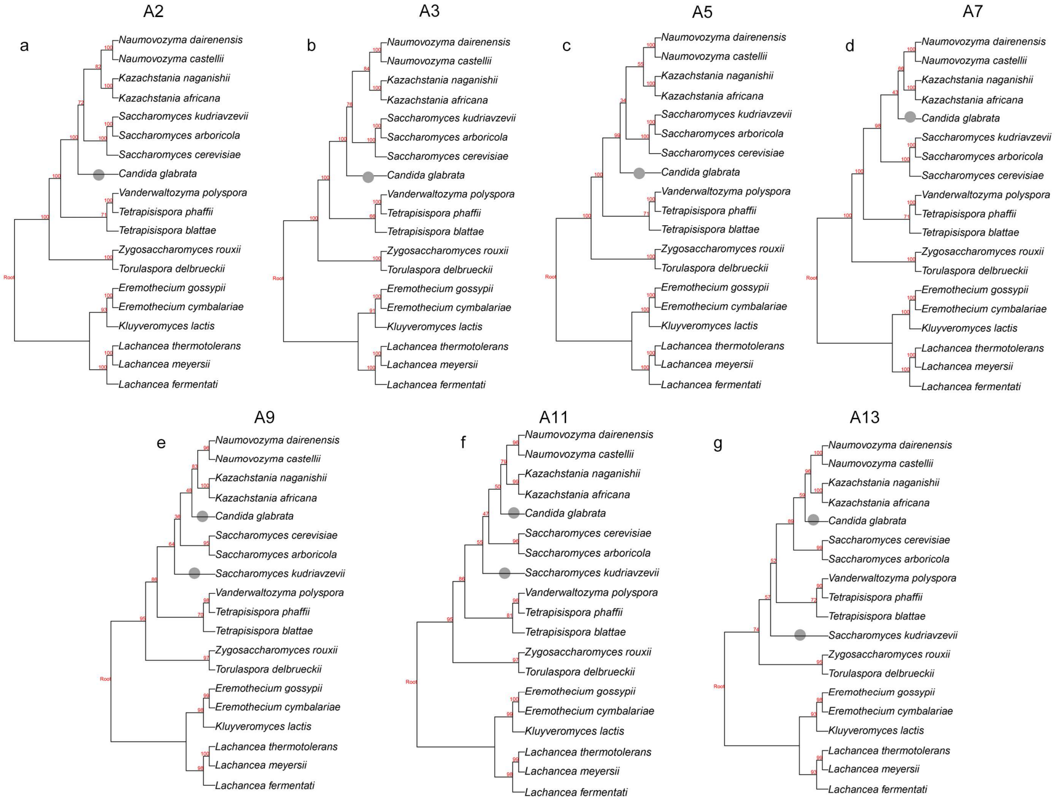

Supplementary Fig. 6 Reconstructed phylogenetic trees of the YGOB data set under seven settings of A_{min}, using the Syn-MRL approach. Bootstrap values are labeled for each node. Branch lengths are not scaled. Differences to the reported phylogeny are marked by grey dots. (**a**) $A_{min} = 2$. (**b**) $A_{min} = 3$. (**c**) $A_{min} = 5$. (**d**) $A_{min} = 7$. (**e**) $A_{min} = 9$. (**f**) $A_{min} = 11$. (**g**) $A_{min} = 13$.

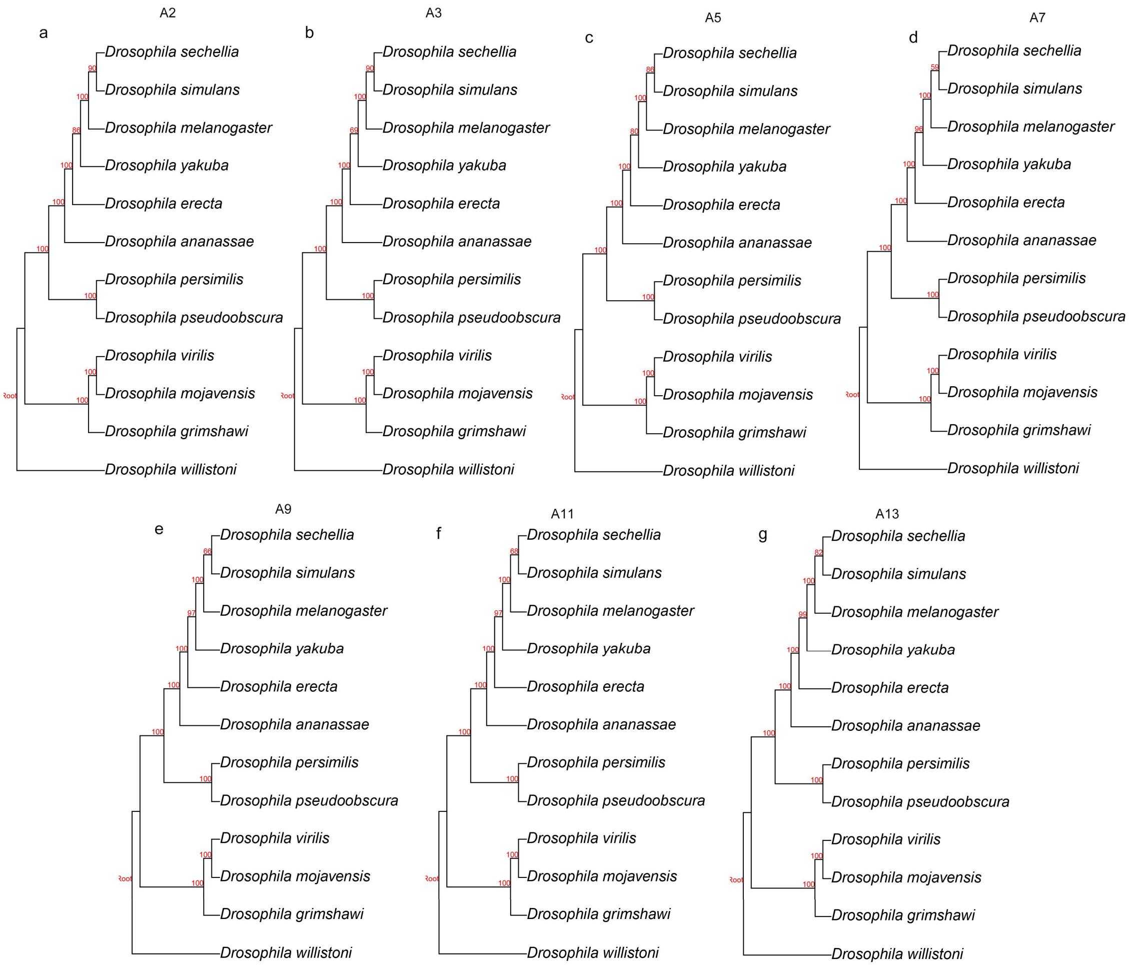

Supplementary Fig. 7 Reconstructed phylogenetic trees of the *Drosophila* data set under seven settings of A_{min}, using the Syn-MRL approach. Bootstrap values are labeled for each node. Branch lengths are not scaled. The tree topologies are consistent across settings and only differ slightly in several bootstrap values. There is one difference compared to the study of Clark et al. 2007, regarding the relationship of *Drosophila yakuba* and *D. erecta*. (**a**) $A_{min} = 2$. (**b**) $A_{min} = 3$. (**c**) $A_{min} = 5$. (**d**) $A_{min} = 7$. (**e**) $A_{min} = 9$. (**f**) $A_{min} = 11$. (**g**) $A_{min} = 13$.

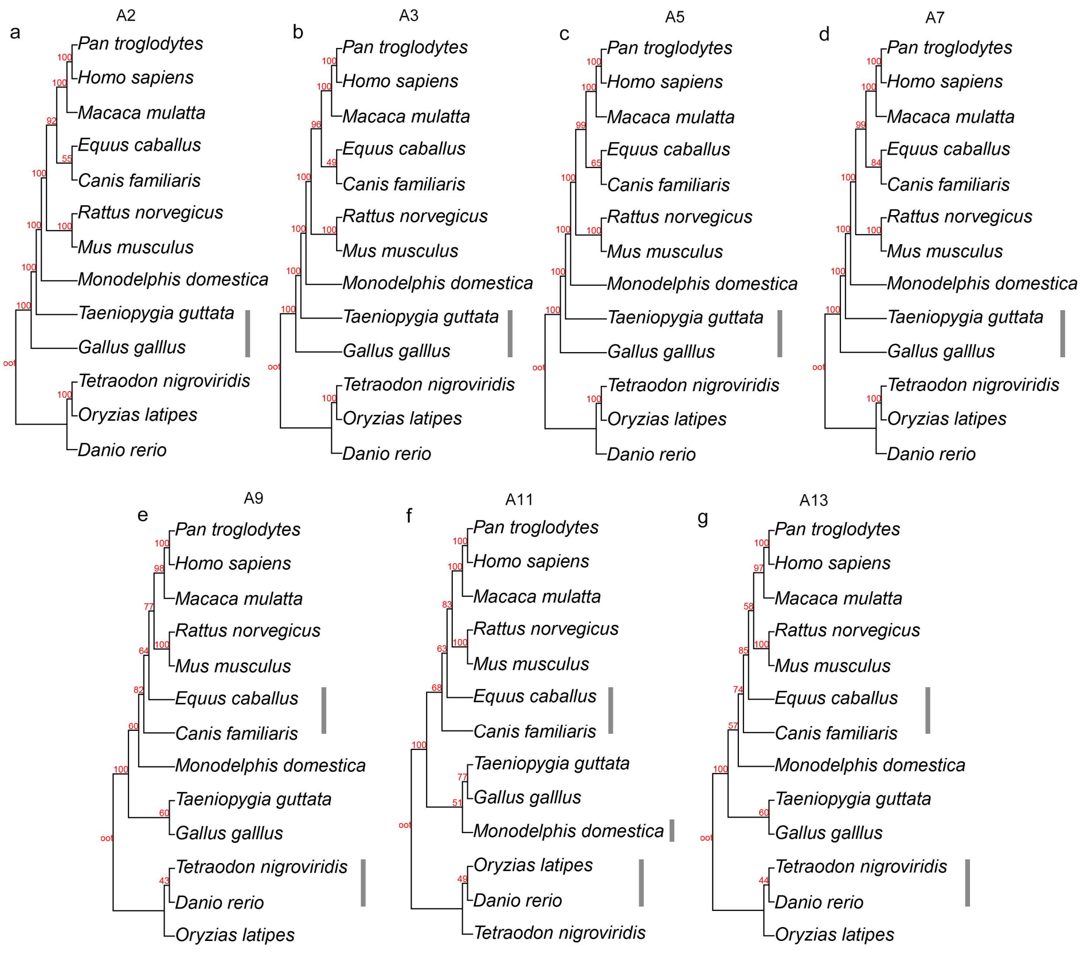

Supplementary Fig. 8 Reconstructed phylogenetic trees of the vertebrate data set under seven settings of A_{min}, using the Syn-MRL approach. Bootstrap values are labeled for each node. Branch lengths are not scaled. Branching patterns that deviate from the consensus phylogeny are labeled by grey bars. (**a**) A_{min} = 2. (**b**) A_{min} = 3. (**c**) A_{min} = 5. (**d**) A_{min} = 7. (**e**) A_{min} = 9. (**f**) A_{min} = 11. (**g**) A_{min} = 13.

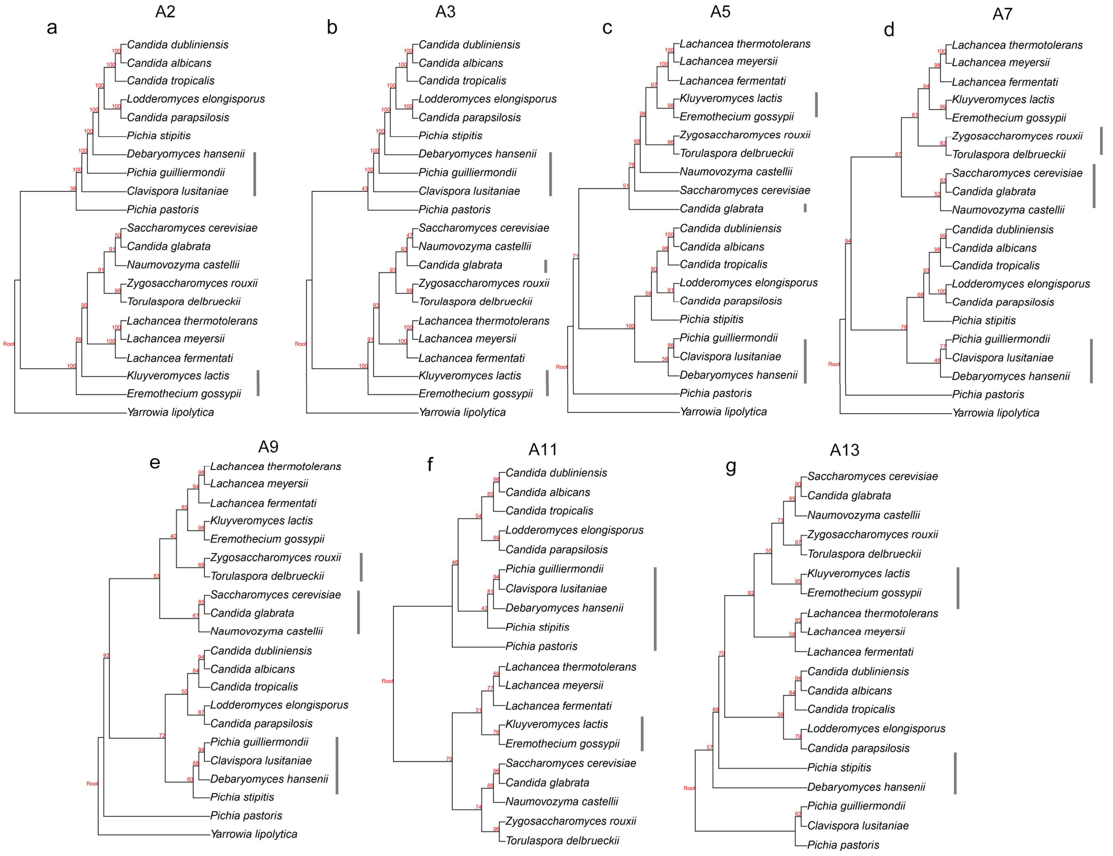

Supplementary Fig. 9 Reconstructed phylogenetic trees of the yeast data set under seven settings of A_{min}, using the Syn-MRL approach. Bootstrap values are labeled for each node. Branch lengths are not scale. Unreliable branching patterns and groups are labeled by grey bars. (**a**) A_{min} = 2. (**b**) A_{min} = 3. (**c**) A_{min} = 5. (**d**) A_{min} = 7. (**e**) A_{min} = 9. (**f**) A_{min} = 11. (**g**) A_{min} = 13.

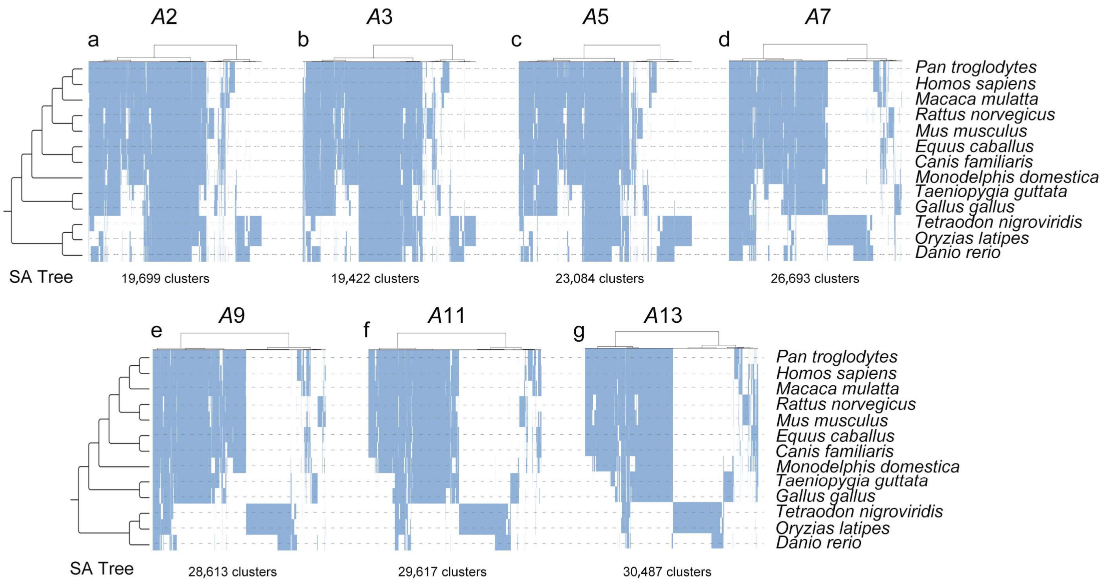

Supplementary Fig. 10 Binary data matrices and clustering of all synteny clusters of the vertebrate dataset under each of the seven settings of A_{min}. Each column represents species compositions of a synteny cluster, in a manner of present (blue) and absence (white) patterns. A sequence alignment-based tree topology was used as a guide on the far left. (**a**) A_{min} = 2. (**b**) A_{min} = 3. (**c**) A_{min} = 5. (**d**) A_{min} = 7. (**e**) A_{min} = 9. (**f**) A_{min} = 11. (**g**) A_{min} = 13.

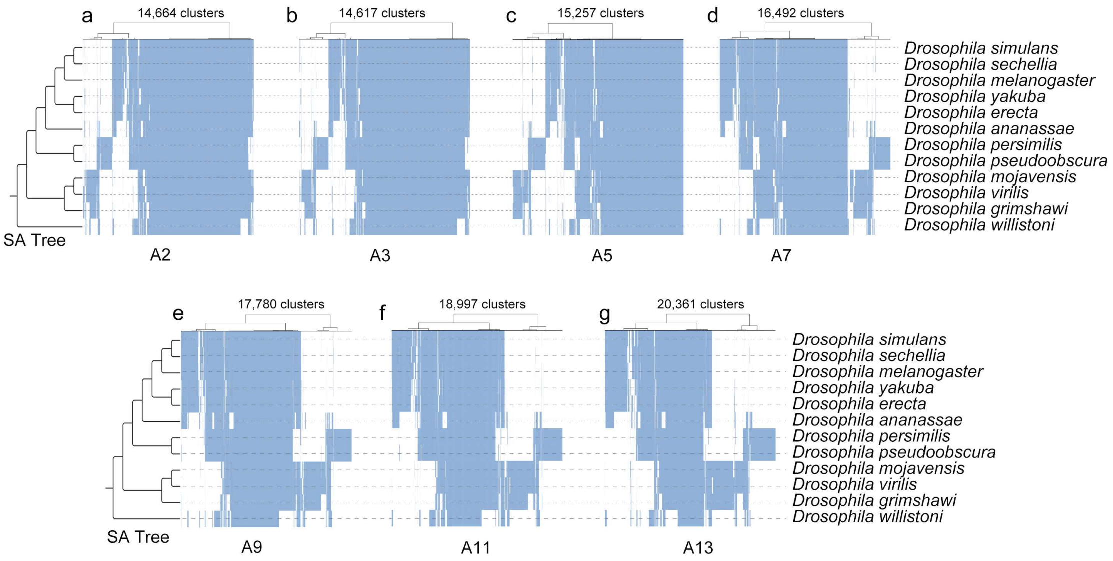

Supplementary Fig. 11 Binary data matrices and clustering of all synteny clusters of the *Drosophila* dataset under each of the seven settings of A_{min}. Each column represents species compositions of a synteny cluster, in a manner of present (blue) and absence (white) patterns. A sequence alignment-based tree topology was used as a guide on the far left. (**a**) $A_{min} = 2$. (**b**) $A_{min} = 3$. (**c**) $A_{min} = 5$. (**d**) $A_{min} = 7$. (**e**) $A_{min} = 9$. (**f**) $A_{min} = 11$. (**g**) $A_{min} = 13$.

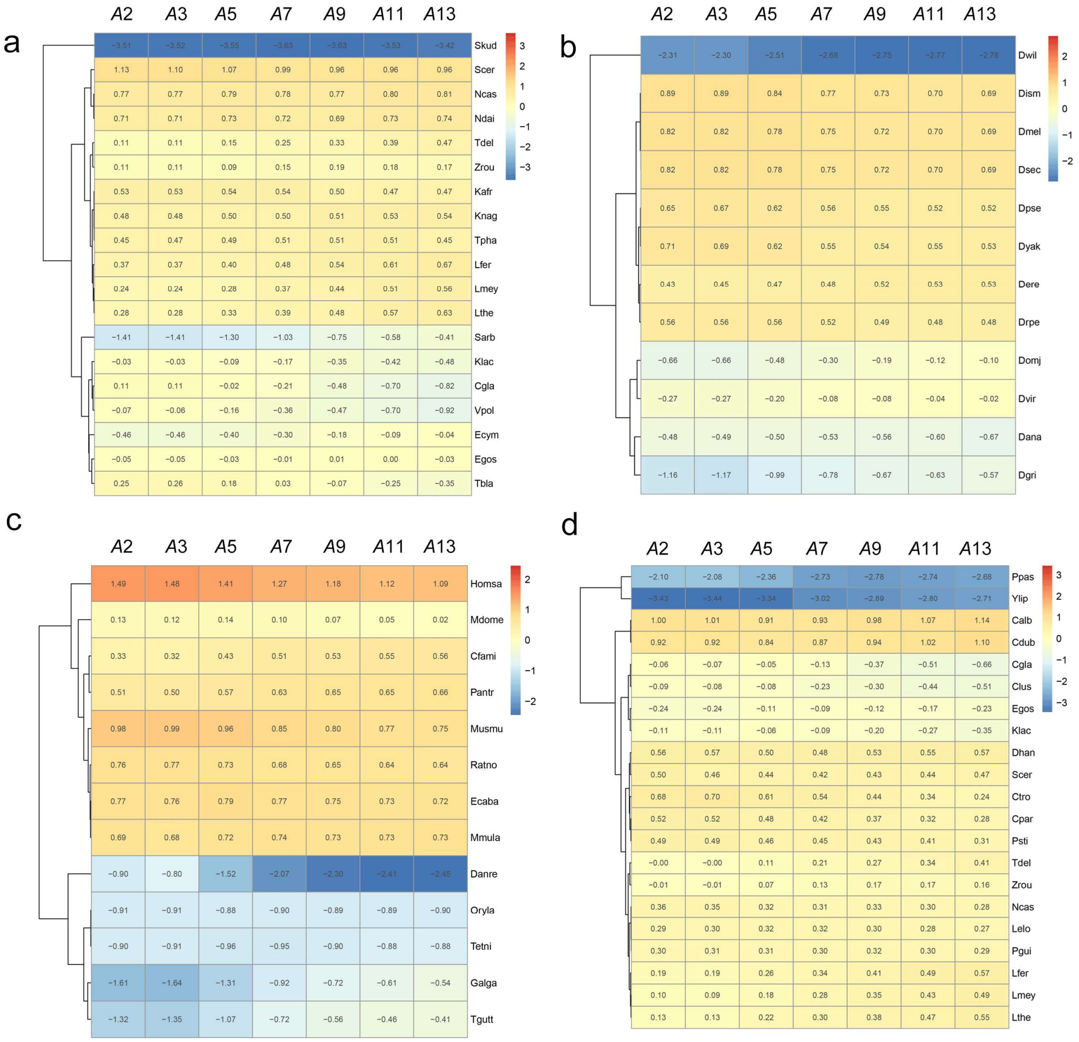

Supplementary Fig. 12 *Z*-score matrices for node percentages (the proportion of nodes for a specific taxon in the syntenic network) of each species for the data sets of (**a**) YGOB, (**b**) *Drosophila*, (**c**) vertebrate, and (**d**) yeast, under each of the seven settings of A_{min}. For the vertebrate data set, the total bird nodes are underrepresented at permissive settings (e.g. A_2 and A_3), whereas fish nodes are underrepresented at stricter settings (e.g. A_7 onwards).

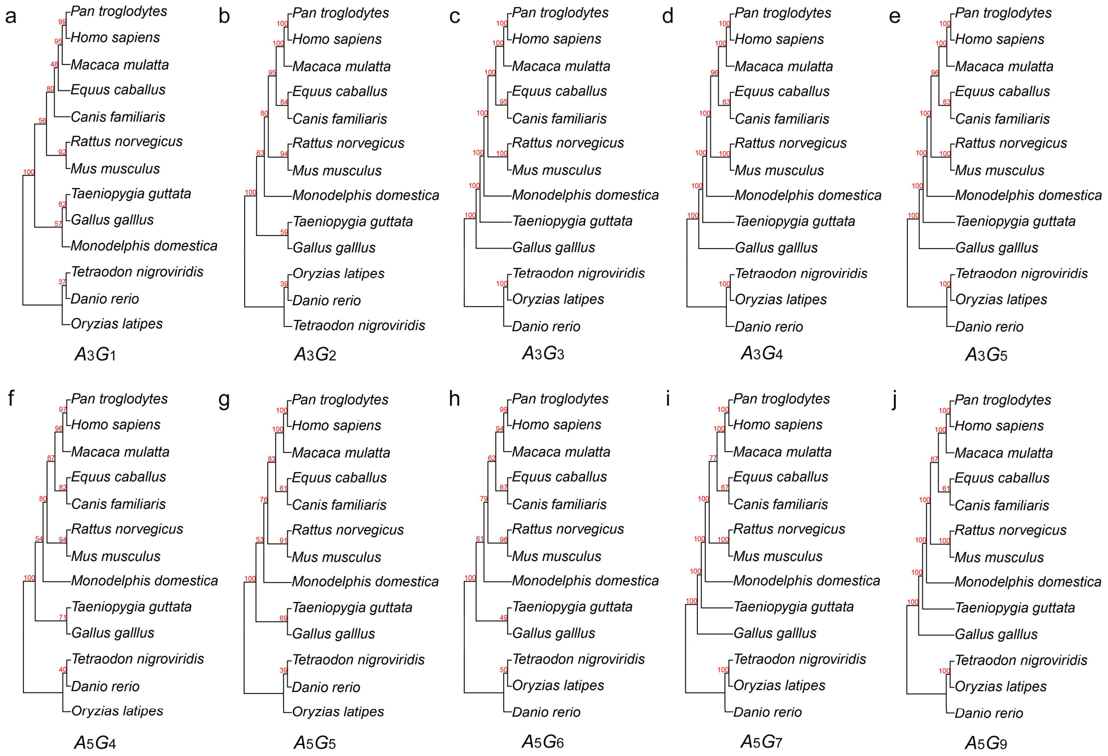

Supplementary Fig. 13 Reconstructed phylogenetic trees of the vertebrate data set under ten settings of combinations of $A_{\min}$ and $G_{\max}$, using the Syn-MRL approach. Bootstrap values are labeled for each node. Branch lengths are not scale. The proper grouping of the fish genomes (which requires a more tolerant setting for this data set) often contradicts the proper grouping of the bird genomes (which requires a stricter setting for this data set). However both groups can be resolved properly at A_3G_2 and A_5G_6 (both with lower bootstrap support values though). The setting name is indicated at the bottom of each tree. The settings of $A_{\min}G_{\max}$ are (**a**) A_3G_1, (**b**) A_3G_2, (**c**) A_3G_3, (**d**) A_3G_4, (**e**) A_3G_5, (**f**) A_5G_4, (**g**) A_5G_5, (**h**) A_5G_6, (**i**) A_5G_7, and (**j**) A_5G_9.

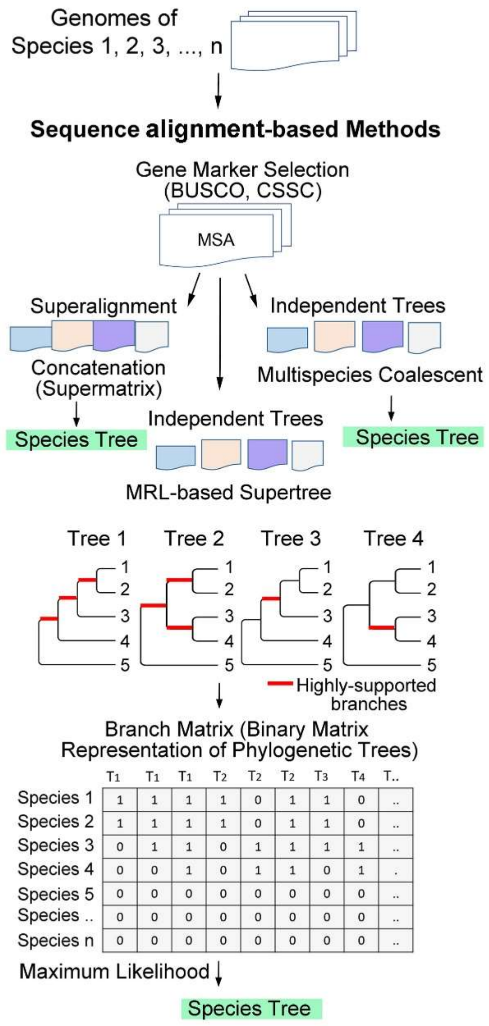

Supplementary Fig. 14 Representative sequence-based methods for reconstructing species trees used in this study, we used superaligment concatenation/supermatrix, multispecies coalescent, and MRL-based supertree methods for two sets of whole-genome-derived markers (BUSCO and CSSC) (see text for details). First, multiple sequence alignments (MSA) were first build for each of the whole-genome-derived markers (BUSCO and CSSC) and used as input for the supermatrix method inferring species trees based on the concatenation of gene alignments. Second, independent gene trees can be inferred for each alignment, after which a species tree can be inferred from the set of obtained gene trees under the multispecies coalescent model, or by using a supertree method. In the latter case, we used a MRL-based method (see text for details). For example, Clade 1+2 is well-supported (BS ≥ 85%) in Tree 1, then this branching order (phylogenetic grouping) is coded as the first column of the matrix, similarly, Clade 1+2+3 of Tree 1 is coded as the second column. Leaves from well-supported nodes of all trees construct a binary branch matrix, which is then used for phylogenetic analysis by maximum likelihood.

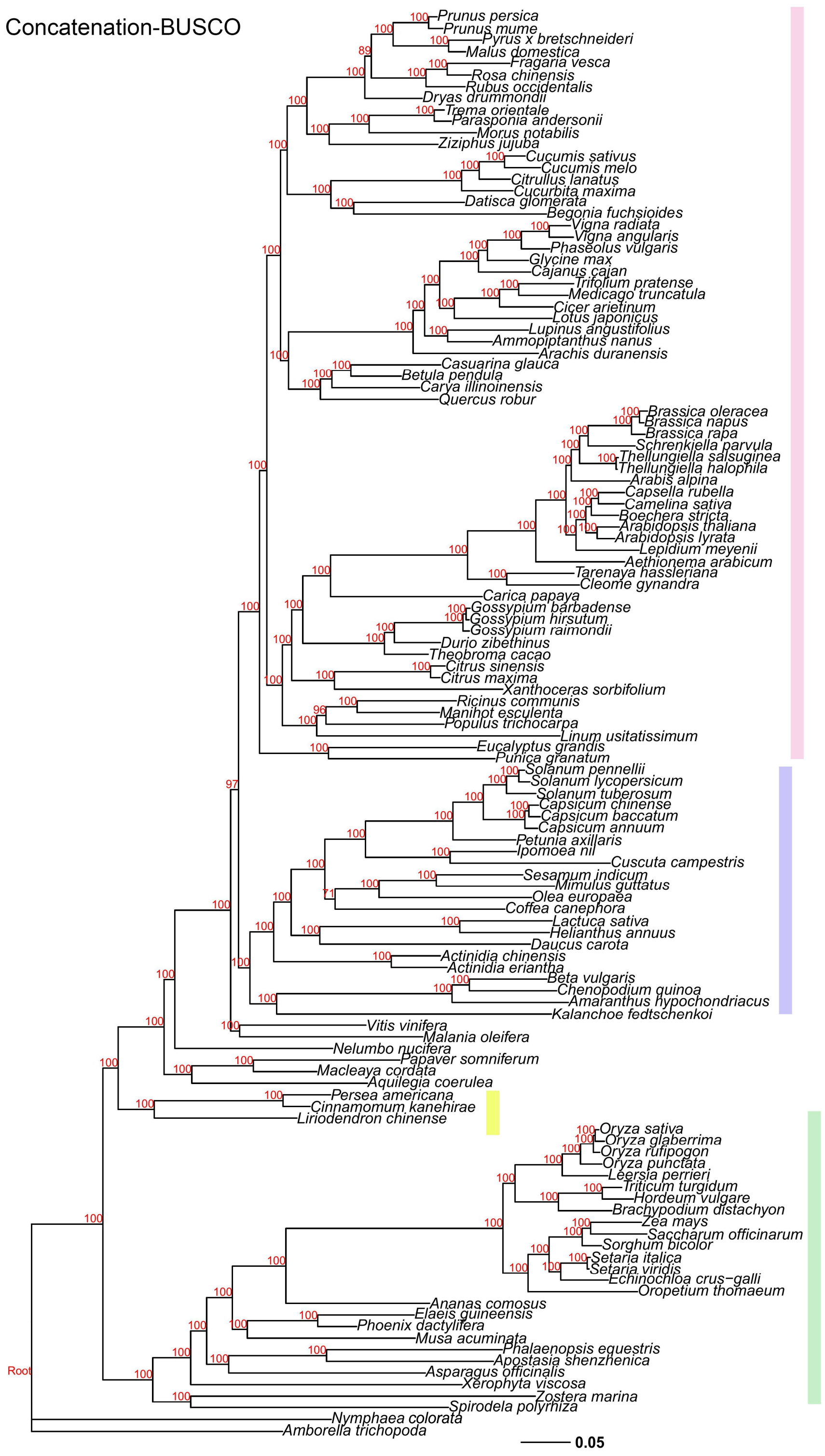

Supplementary Fig. 15 Maximum likelihood concatenation-BUSCO tree. The tree is rooted by *Amborella*, and four main clades, i.e. superrosids, superasterids, monocots, and magnoliids are shaded in light-red, light-purple, light-green, and light-yellow, respectively. This applies to all other phylogenetic trees.

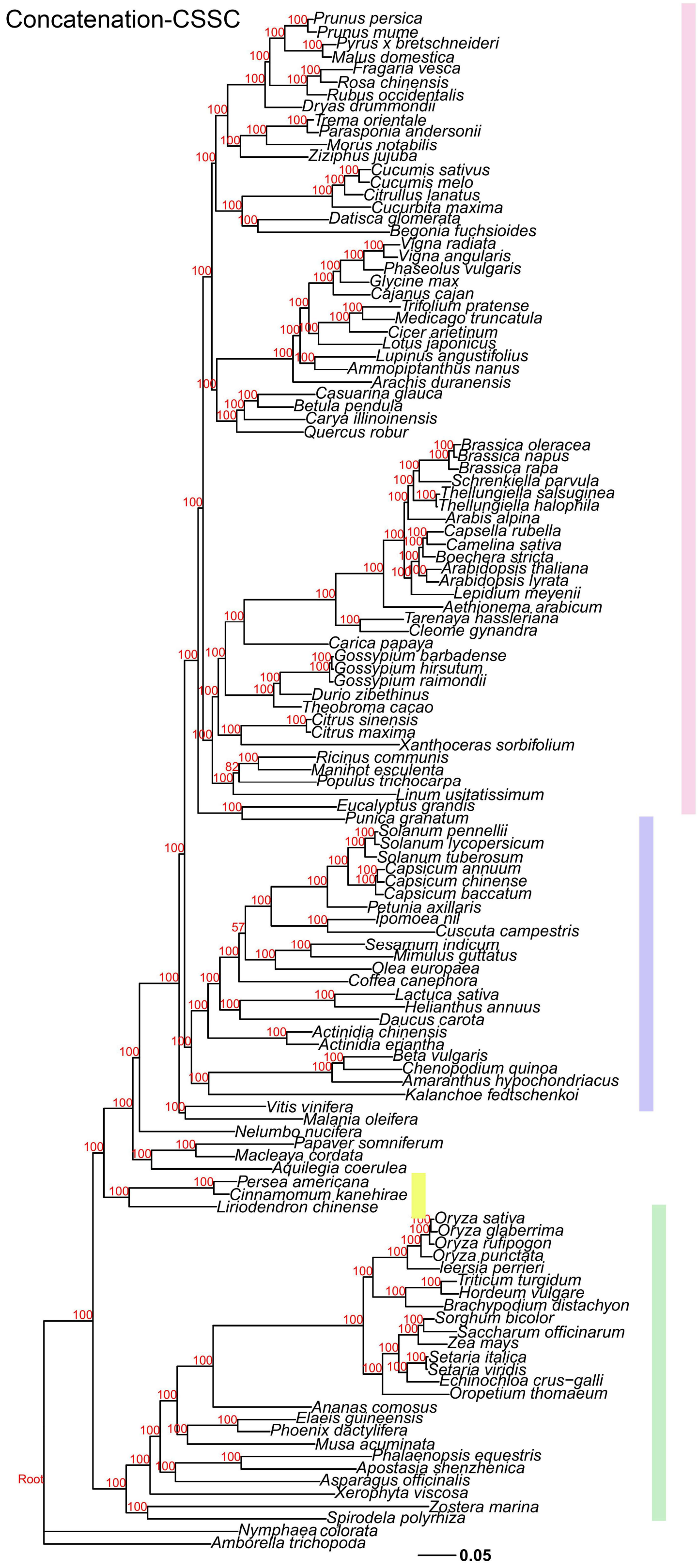

Supplementary Fig. 16 Maximum likelihood concatenation-CSSC tree.

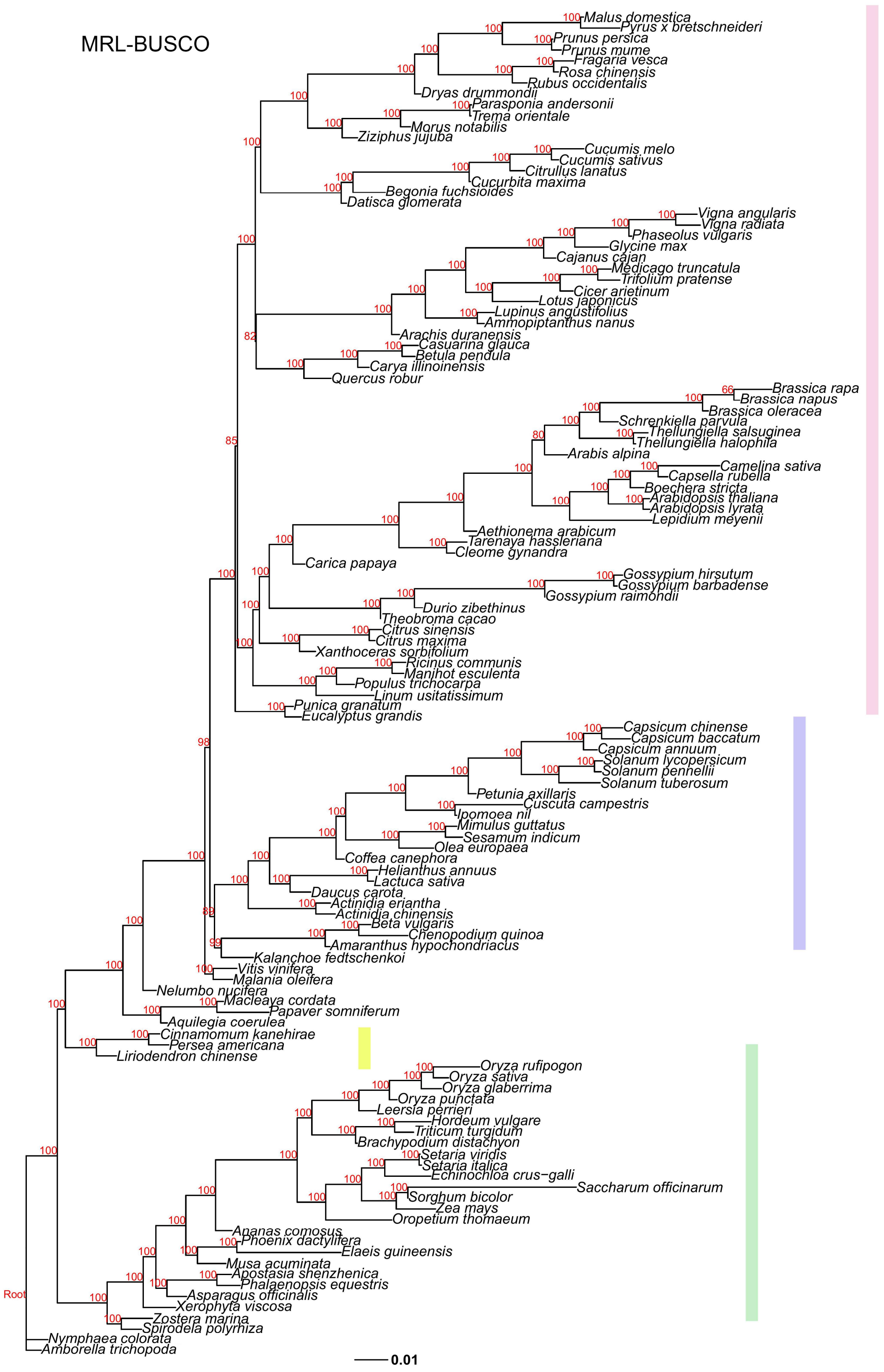

Supplementary Fig. 17 MRL-BUSCO tree.

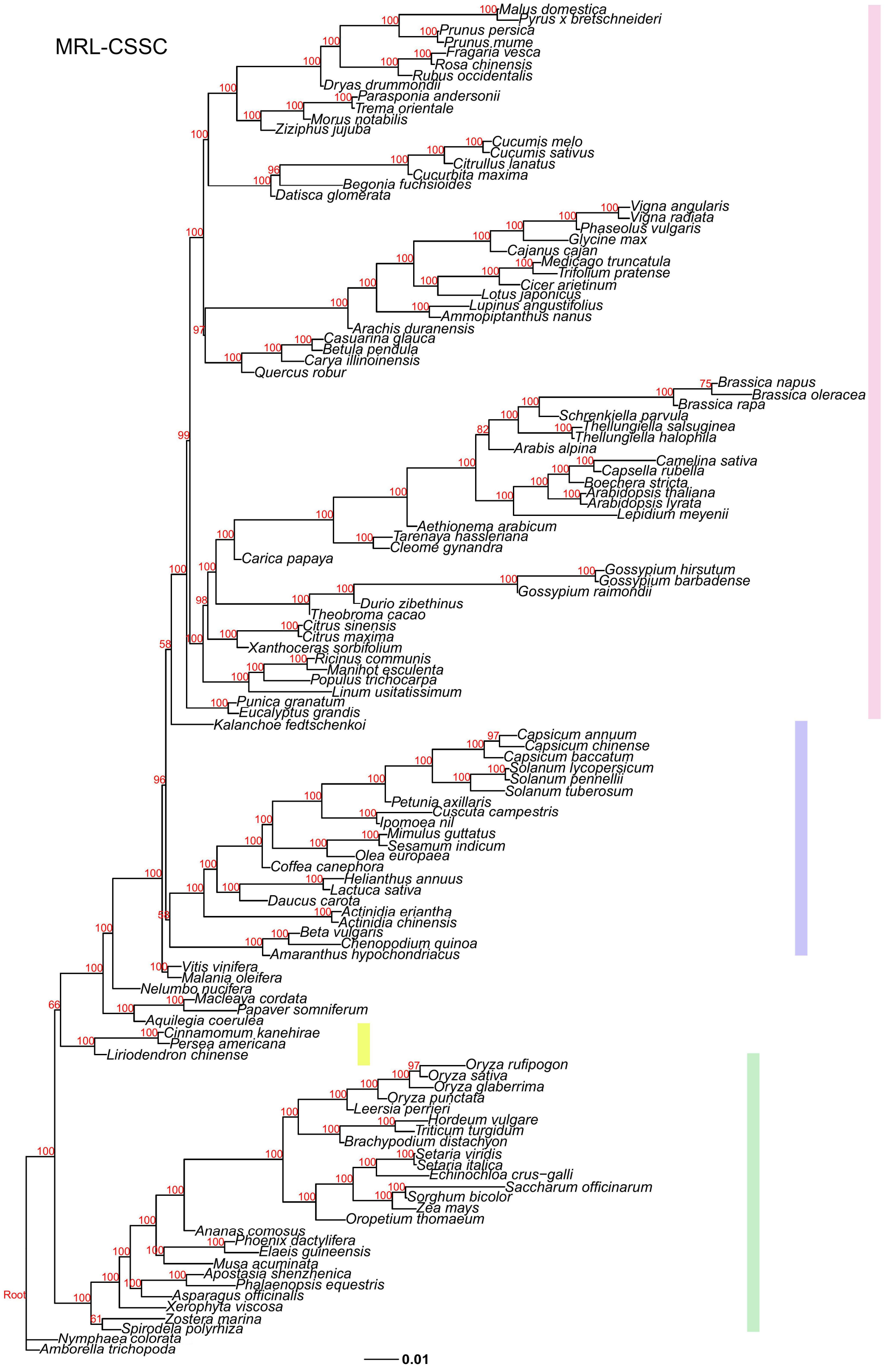

Supplementary Fig. 18 MRL-CSSC tree.

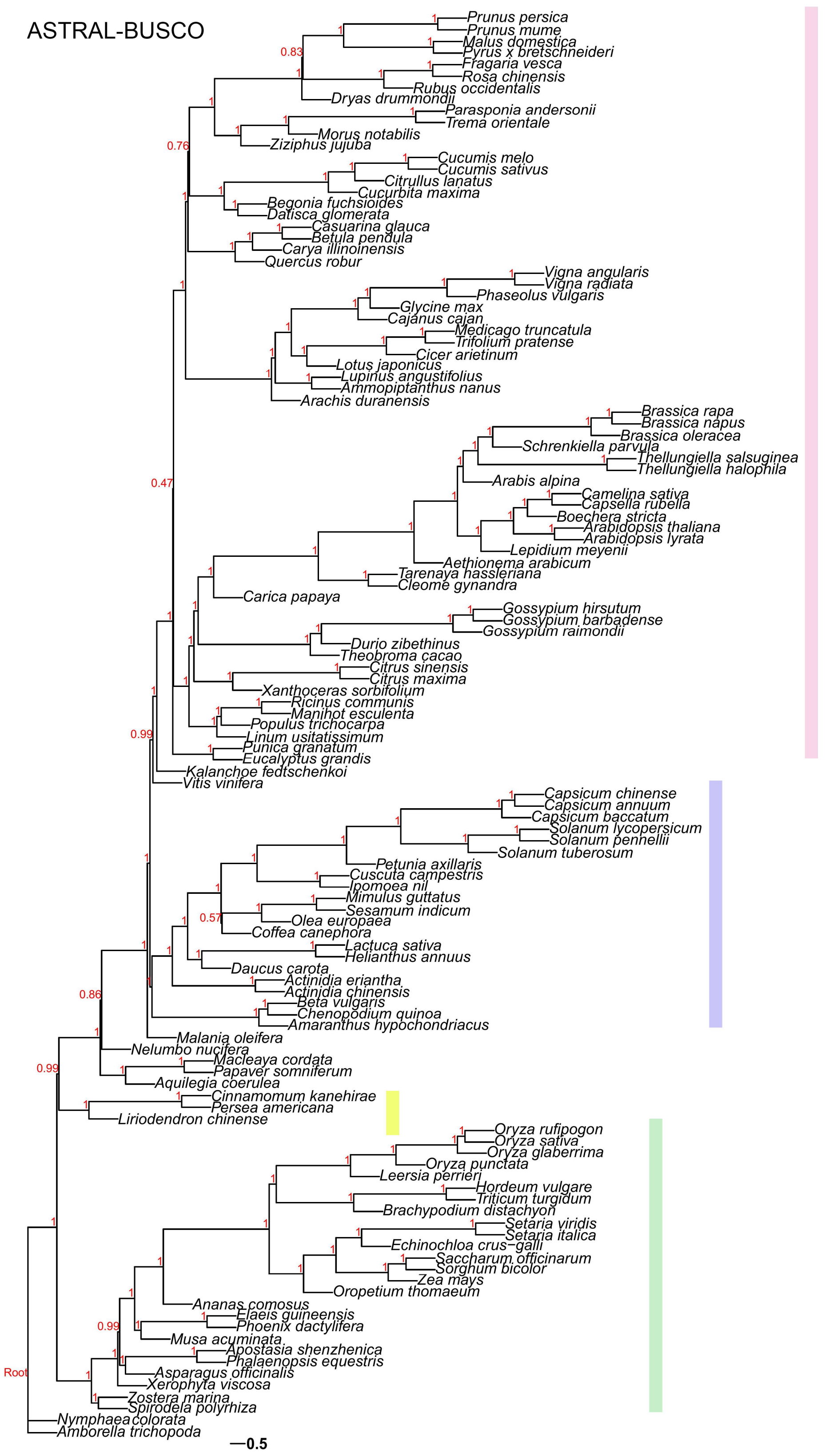

Supplementary Fig. 19 ASTRAL-BUSCO tree.

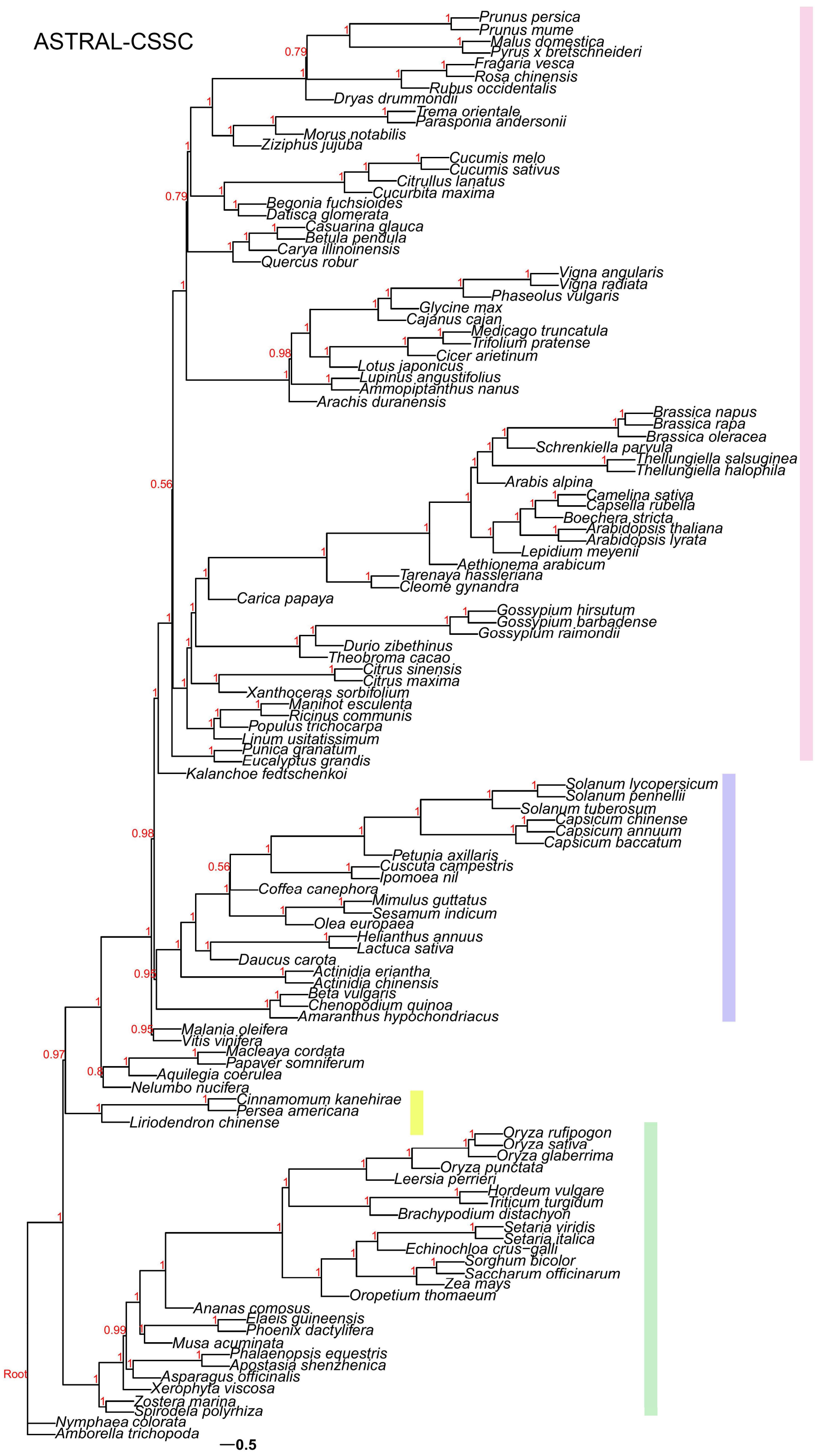

Supplementary Fig. 20 ASTRAL-CSSC tree.

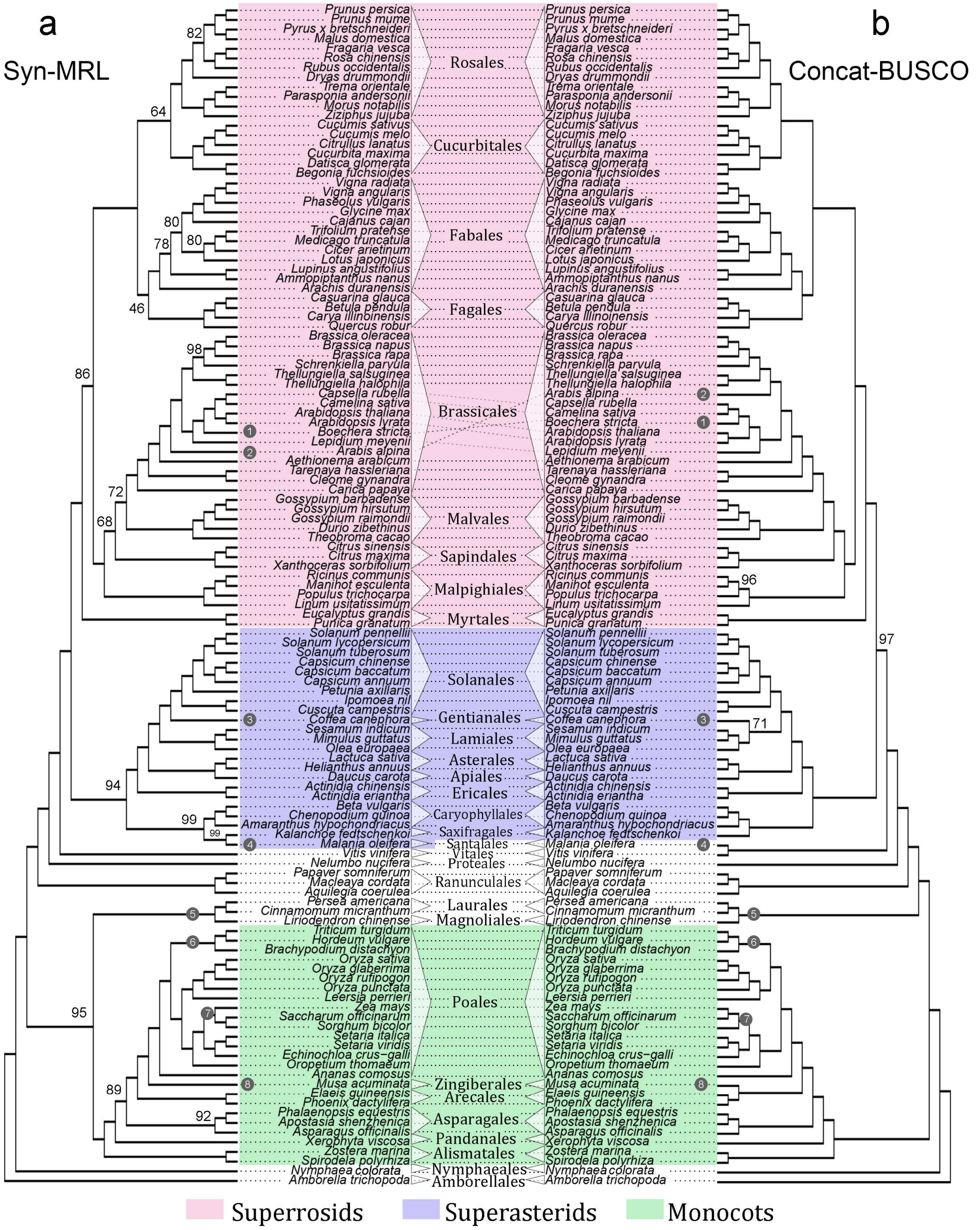

Supplementary Fig. 21 Comparison of (a) synteny tree and (b) the SA tree. Both trees are rooted by *Amborella*, and three main clades, i.e. superrosids, superasterids, and monocots are shaded in light-red, light-purple, and light-green, respectively. Eight differences are indicated by indexed black dots. Branches are not drawn to scale. Ultrafast bootstrapping values (see text for details) were marked for nodes with less than 100% support.

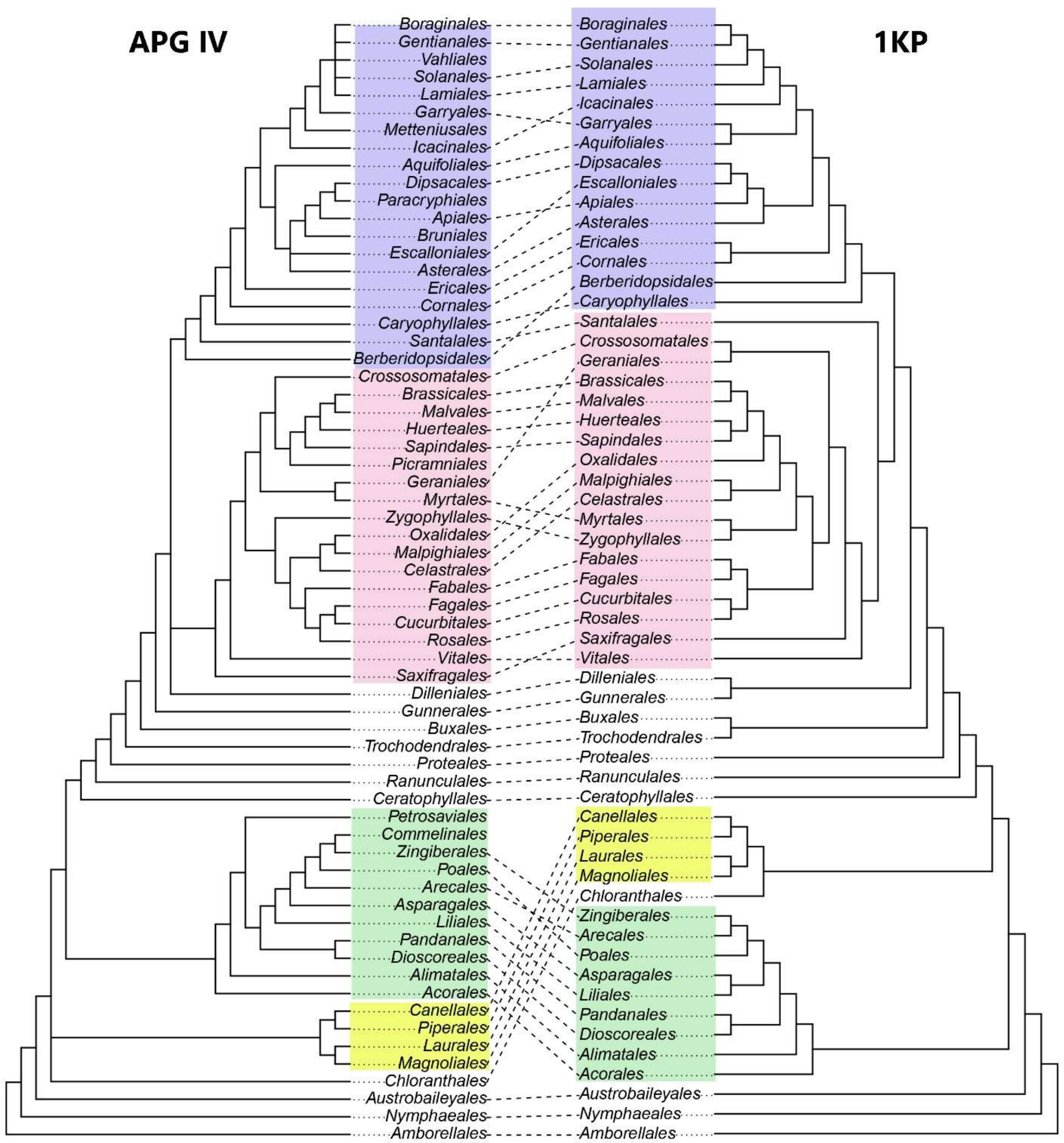

Supplementary Fig. 22 Comparison of phylogenetic relationships between the phylogeny of Angiosperm Phylogeny Group IV and 1KP.

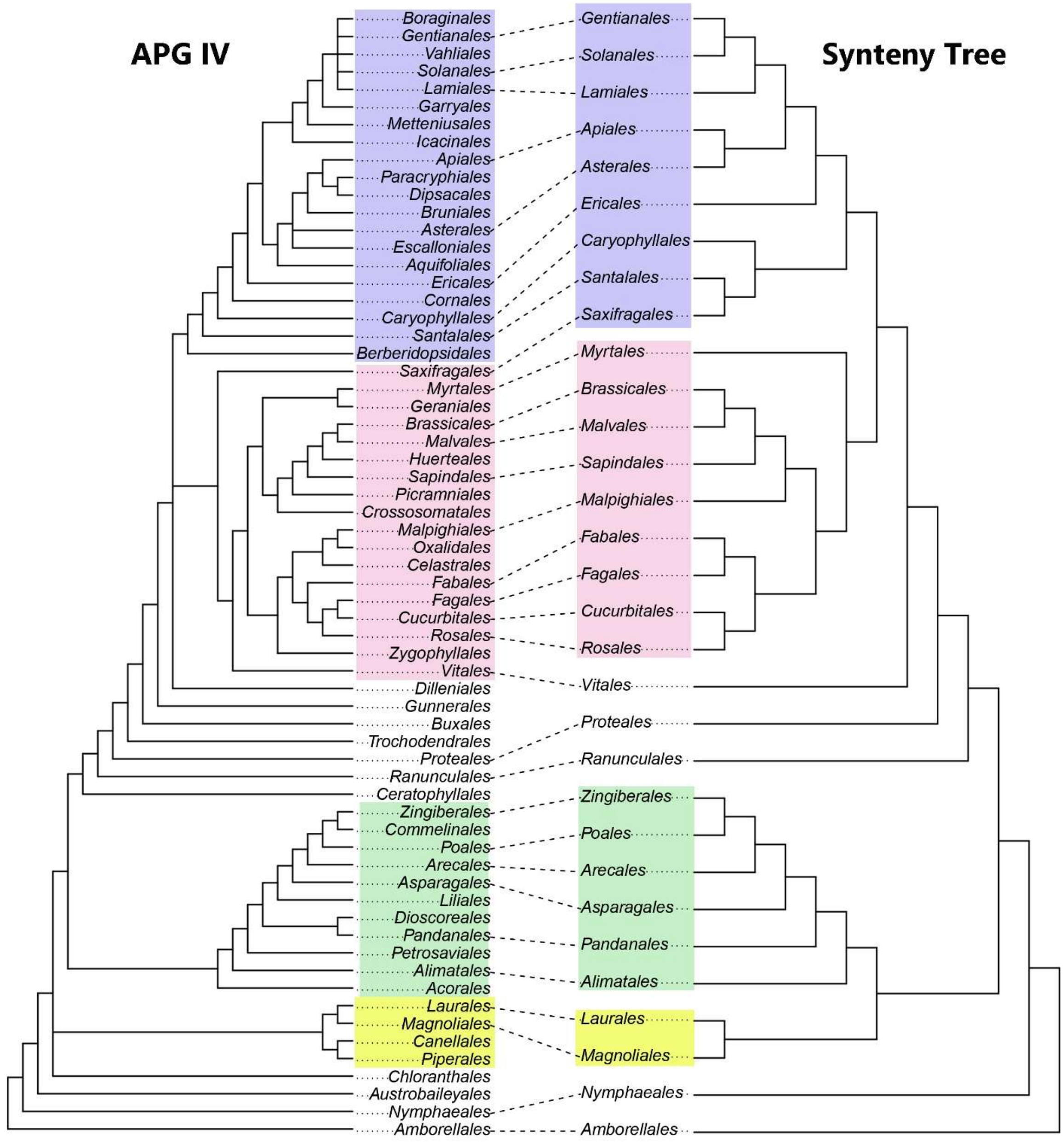

Supplementary Fig. 23 Comparison of phylogenetic relationships between APG IV and the synteny tree.

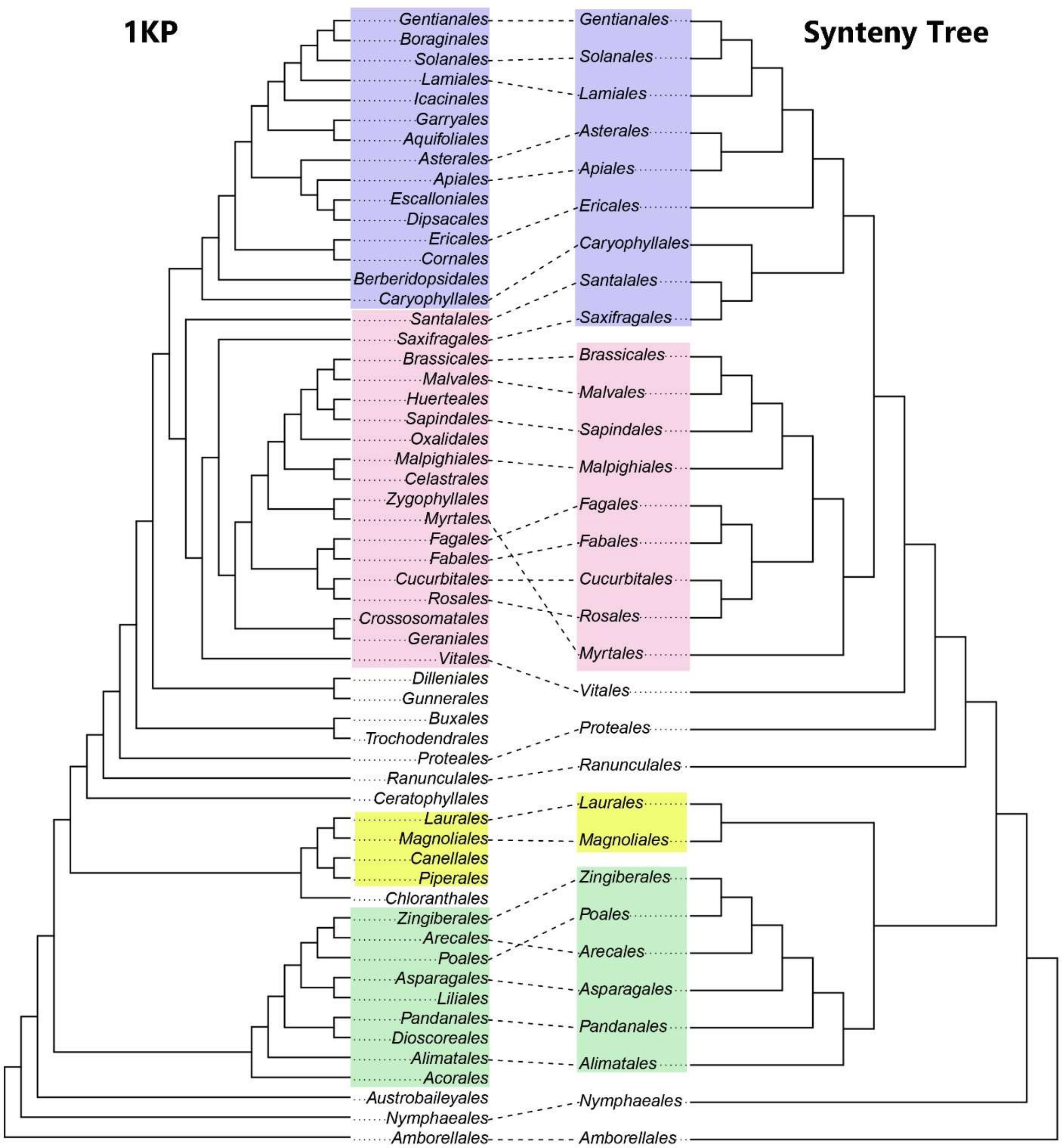

Supplementary Fig. 24 Comparison of phylogenetic relationships between the phylogeny of 1KP study (angiosperms part) and the synteny tree.

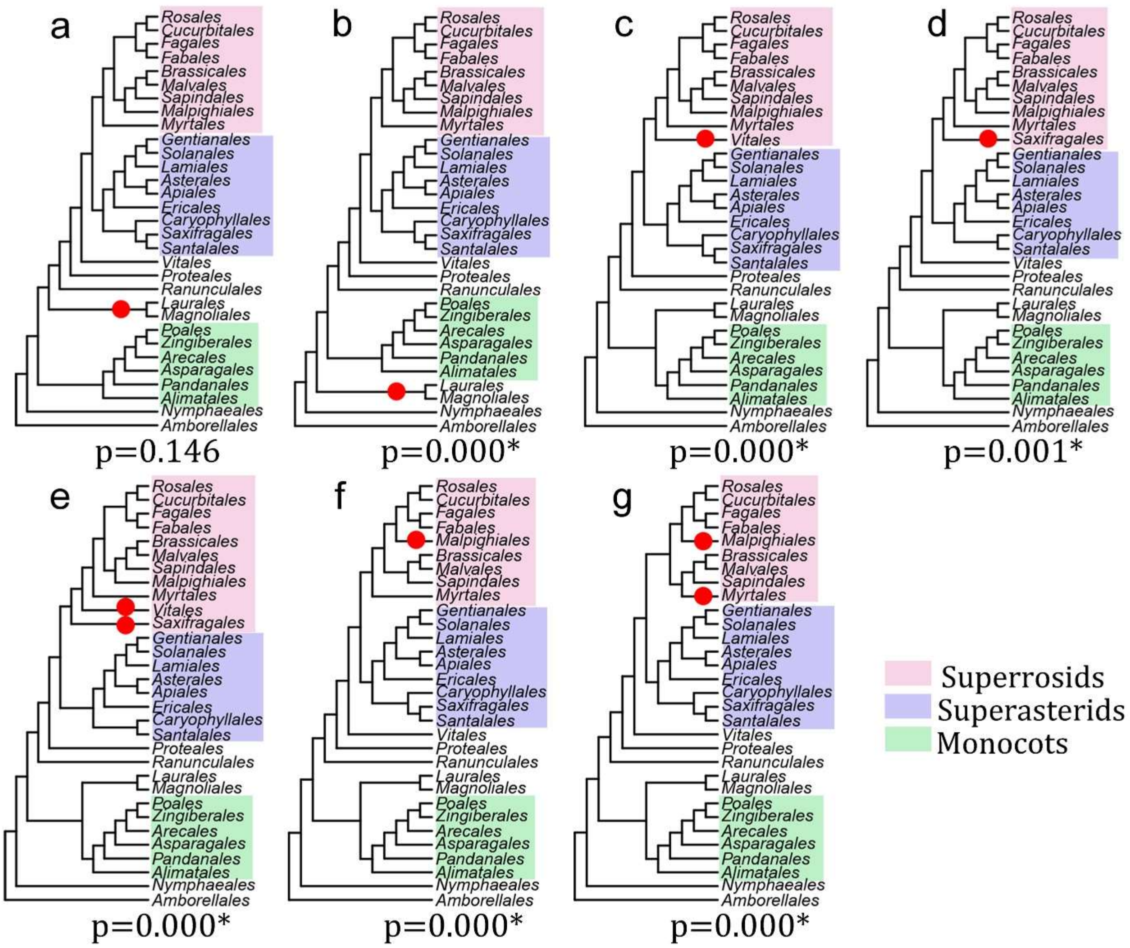

Supplementary Fig. 25 Approximate unbiased (AU) test for alternative topologies, with resulting *p* values (one-sided) indicated under the trees. Alternative (tested) topologies include (**a**) magnoliids as sister to eudicots, which is the only scenario tested that cannot be significantly rejected (see text for details). (**b**) magnoliids as sister to both monocots and eudicots, (**c**) Vitales as early-diverging rosids, (**d**) Saxifragales as early-diverging rosids, (**e**) Vitales and Saxifragales as early-diverging rosids, (**f**) Malpighiales as early-diverging Fabids, and (**g**) Malpighiales as early-diverging Fabids plus Myrtales as early-diverging Malvids.

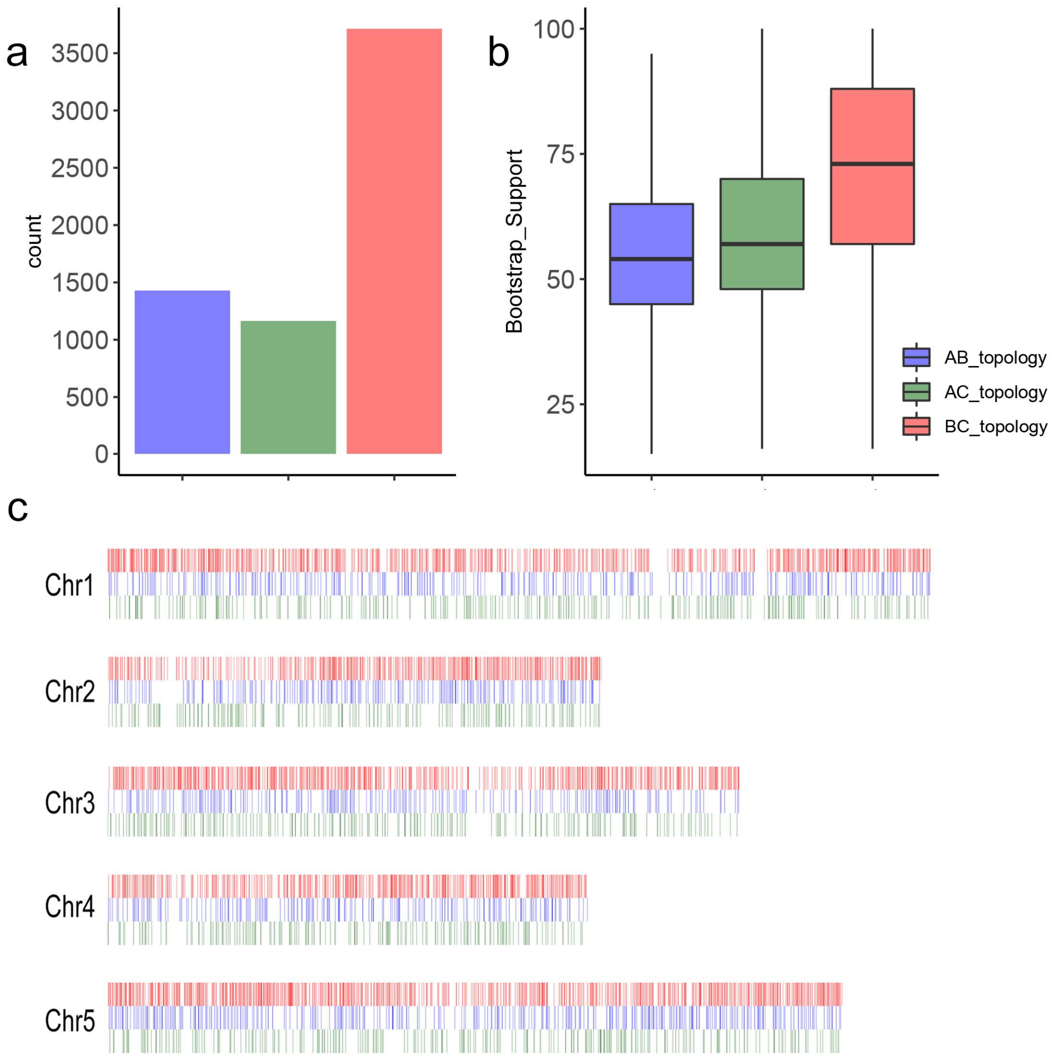

Supplementary Fig. 26 Tree topologies of the filtered gene trees based on low-copy gene markers of the ABC dataset. (a) Number of trees supporting different topologies. (b) Bootstrap supports of the nodes supporting the branching order. The boxplots indicate the minimum, maximum, median (the middle hinge), first quartile (the lower hinge), and third quartile (the upper hinge) in the data sets (n = 1429, 1164, and 3713 for the AB_topology, AC_topology, and BC_topology). The whiskers represent the 1.5 inter-quartile range (IQR) extending from the hinges. (c) Distribution of the tree topologies across *Arabidopsis thaliana* genome.

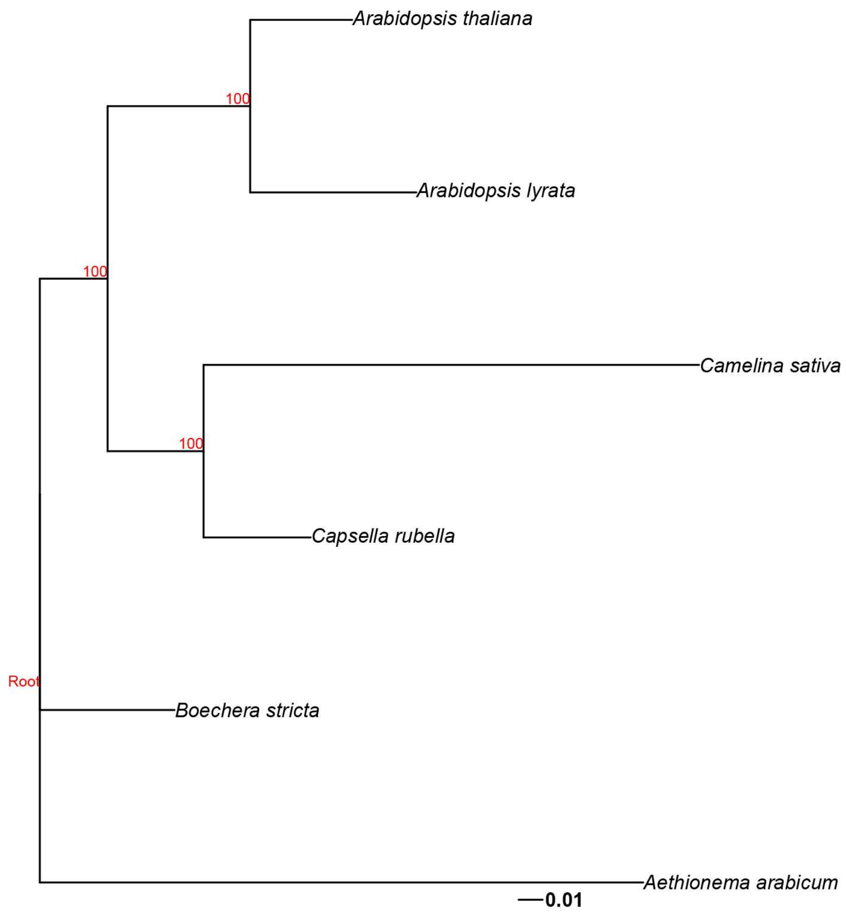

Supplementary Fig. 27 Reconstructed species tree for the ABC data set using Syn-MRL.

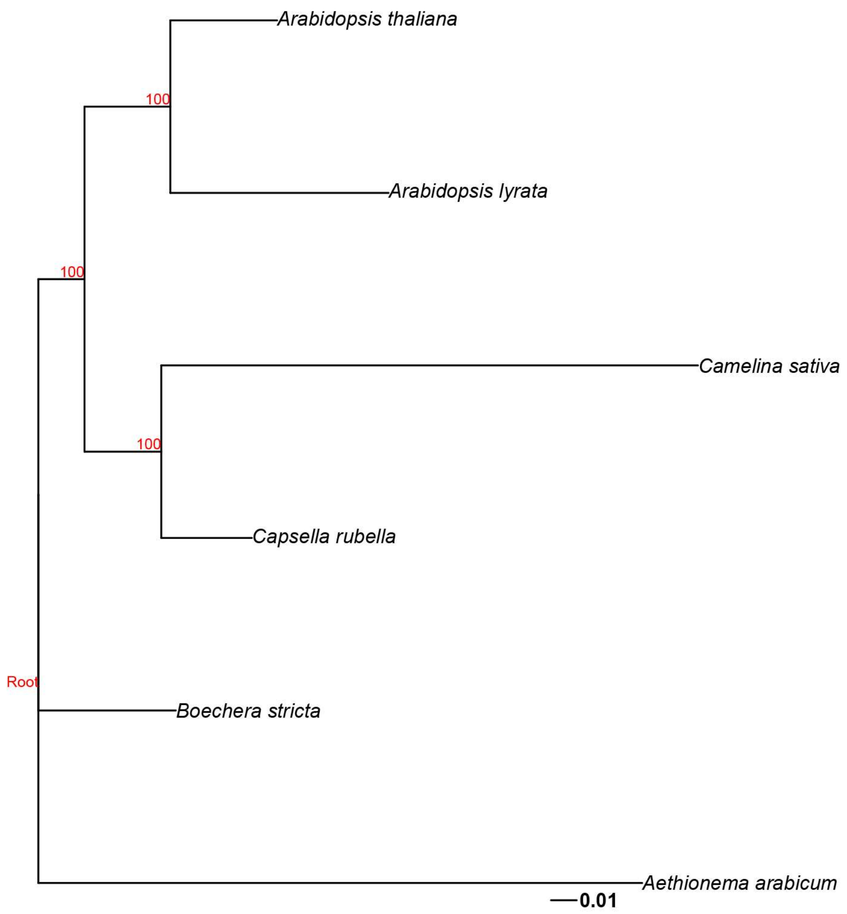

Supplementary Fig. 28 Reconstructed species tree for the ABC data set based on orthogroup gene content.

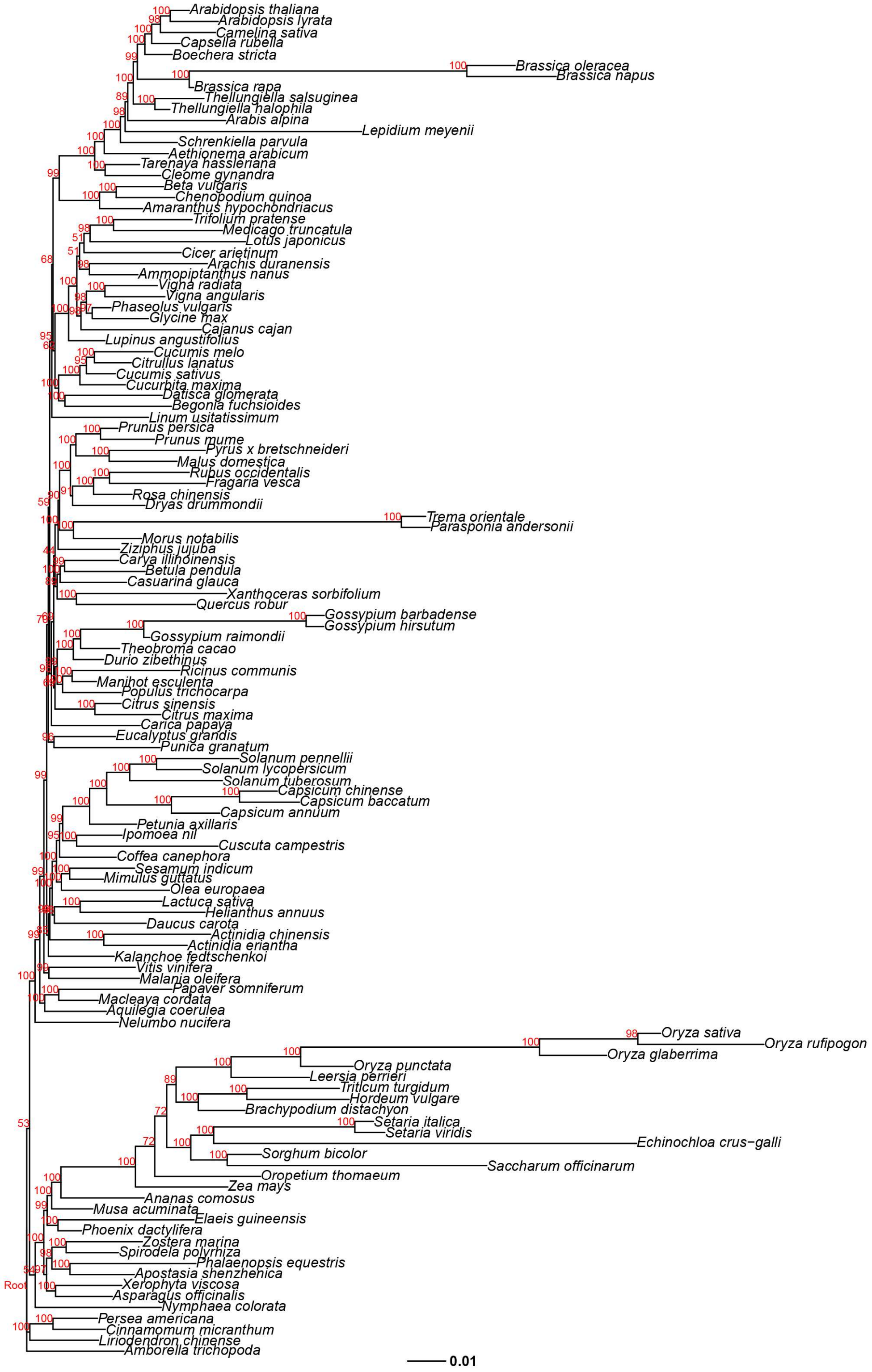

Supplementary Fig. 29 Reconstructed species tree for the 123 plant genomes based on orthogroup gene content.

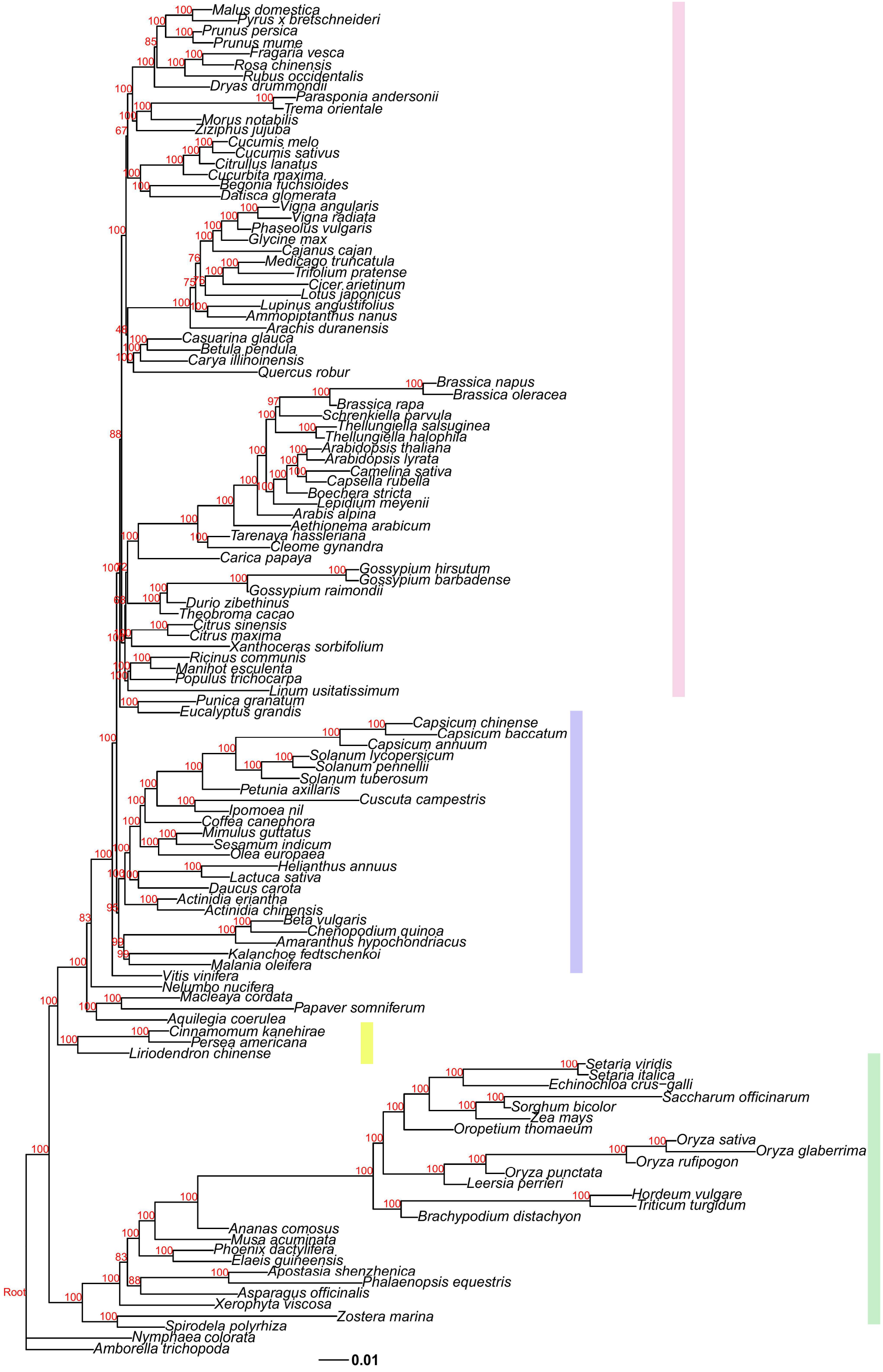

Supplementary Fig. 30 Syn-MRL tree based on the synteny matrix without 1107 (Figure 6a) specific signals.
