# Supplementary material for: Whole-genome microsynteny-based phylogeny of angiosperms
Source: Nat Commun. 2021 Jun 9;12:3498. doi: 10.1038/s41467-021-23665-0 (PMC8190143; doi:10.1038/s41467-021-23665-0)
Supplement: Supplementary file 3 — Description of Additional Supplementary Files [file 41467_2021_23665_MOESM3_ESM.pdf]

Description of Additional Supplementary Files

File Name: Supplementary Data 1
Description: Genome resources for the YGOB, *Drosophila*, Vertebrate, and yeast data sets.

File Name: Supplementary Data 2
Description: List of plant genomes used in this study.

File Name: Supplementary Data 3
Description: Gene trees and topology analysis of the low-copy gene orthogroups of the ABC data set.

File Name: Supplementary Data 4
Description: Unassigned singleton genes characterized by OrthoFinder compared to the synteny network clustering for the ABC data set.

File Name: Supplementary Data 5
Description: Phylogenomic profile matrix rearranged by *Cinnamomum kanehirae* chromosomes and gene orders.
