# Supplementary material for: Whole-genome microsynteny-based phylogeny of angiosperms
Source: Nat Commun. 2021 Jun 9;12:3498. doi: 10.1038/s41467-021-23665-0 (PMC8190143; doi:10.1038/s41467-021-23665-0)
Supplement: Supplementary file 9 — Reporting Summary [file 41467_2021_23665_MOESM9_ESM.pdf]

nature research

Corresponding author(s): Yves Van de Peer, Tao Zhao

Last updated by author(s): May 6, 2021

Reporting Summary

Nature Research wishes to improve the reproducibility of the work that we publish. This form provides structure for consistency and transparency in reporting. For further information on Nature Research policies, see our Editorial Policies and the Editorial Policy Checklist.

Statistics

For all statistical analyses, confirm that the following items are present in the figure legend, table legend, main text, or Methods section.

n/a	Confirmed	
☐	☒	The exact sample size (*n*) for each experimental group/condition, given as a discrete number and unit of measurement
☒	☐	A statement on whether measurements were taken from distinct samples or whether the same sample was measured repeatedly
☐	☒	The statistical test(s) used AND whether they are one- or two-sided<br>*Only common tests should be described solely by name; describe more complex techniques in the Methods section.*
☒	☐	A description of all covariates tested
☐	☒	A description of any assumptions or corrections, such as tests of normality and adjustment for multiple comparisons
☐	☒	A full description of the statistical parameters including central tendency (e.g. means) or other basic estimates (e.g. regression coefficient) AND variation (e.g. standard deviation) or associated estimates of uncertainty (e.g. confidence intervals)
☐	☒	For null hypothesis testing, the test statistic (e.g. *F*, *t*, *r*) with confidence intervals, effect sizes, degrees of freedom and *P* value noted<br>*Give P values as exact values whenever suitable.*
☒	☐	For Bayesian analysis, information on the choice of priors and Markov chain Monte Carlo settings
☒	☐	For hierarchical and complex designs, identification of the appropriate level for tests and full reporting of outcomes
☒	☐	Estimates of effect sizes (e.g. Cohen's *d*, Pearson's *r*), indicating how they were calculated

Our web collection on statistics for biologists contains articles on many of the points above.

Software and code

Policy information about availability of computer code

Data collection	No software was used in data collection.
Data analysis	Scripts for synteny network construction, network clustering, and phylogenomic profiling are available at Github (http://github.com/zhaotao1987/SynNet-Pipeline). Related code (for preparing the data matrices for phylogenetic reconstructions) and software parameters are available at Github (http://github.com/zhaotao1987/Syn-MRL). The gene family syntenic network simulation program is available at Github (http://github.com/arzwa/TaoNet). All other data analyses were performed using freely available softwares. Namely, DIAMOND (version v0.9.18.119), MCScanX, Infomap(version 0.20.0), IQ-TREE (version 1.7-beta7), OrthoFinder (version 2.4.0), MAFFT (version 7.187), trimAl (version v1.4.rev15), catfasta2phyml, BUSCO (v3.0), ASTRAL-Pro, DeadBird julia package, R, Python, Julia, and Perl. Specific parameters used are provided in the methods.

For manuscripts utilizing custom algorithms or software that are central to the research but not yet described in published literature, software must be made available to editors and reviewers. We strongly encourage code deposition in a community repository (e.g. GitHub). See the Nature Research guidelines for submitting code & software for further information.

Data

Policy information about availability of data

All manuscripts must include a data availability statement. This statement should provide the following information, where applicable:

- Accession codes, unique identifiers, or web links for publicly available datasets
- A list of figures that have associated raw data
- A description of any restrictions on data availability

Data sets used in this study are available at DataVerse (http://doi.org/10.7910/DVN/7ZZWIH). This includes all annotated protein sequences in FASTA format of each genome, entire synteny network database (edge list), network clustering result, trimmed alignments and corresponding phylogenetic trees of BUSCO and CSSC

genes, bipartitions with support values for each tree, and binary data matrices.

Field-specific reporting

Please select the one below that is the best fit for your research. If you are not sure, read the appropriate sections before making your selection.

☐ Life sciences ☐ Behavioural & social sciences ☒ Ecological, evolutionary & environmental sciences

For a reference copy of the document with all sections, see nature.com/documents/nr-reporting-summary-flat.pdf

Ecological, evolutionary & environmental sciences study design

All studies must disclose on these points even when the disclosure is negative.

Study description	We have reconstructed the phylogenetic relationships of flowering plants based on gene order arrangements. Fully-sequenced quality angiosperm genomes were used for the analysis.
Research sample	One hundred and twenty-three high quality plant genomes were used for the study, representing 52 different plant families and 31 plant orders in angiosperms. The source of these 123 plant genomes and sampling strategy are described below in 'Sampling strategy'. Moreover, we used empirical data sets to benchmark our method. This includes genomes of yeasts (used in YGOB (Byrne et al., 2005), and Drillion et al., 2020), Drosophila (Clark et al., 2007), and vertebrates (Drillon et al., 2020).
Sampling strategy	Reference genomes were obtained from public repositories, including Phytozome, CoGe, GigaDB, and NCBI. After constructing our synteny network database and clustering, poor quality genomes could be relatively easily identified, and were removed from the database for further analysis. We acknowledge that our taxon sampling is limited to currently available high-quality genomes and thus many important lineages could not be included. For example, some important plant lineages still lack well-assembled genomes (such as for Dilleniales and Chloranthales), while others only have few representatives (such as Santalales and Saxifragales), and still others are relatively 'over-represented' (such as Brassicales, Fabales, and Poales). For 'over-represented' orders, we kept all qualified genomes because this provides an opportunity to test the resolution and robustness of our approach at different levels (e.g. order-level, family-level, and species-level).
Data collection	For each genome, we downloaded FASTA format files containing protein sequences of all predicted gene models and the genome annotation files (GFF/BED) containing the positions of all the genes. We modified all peptide sequence files and genome annotation GFF/BED files with corresponding species abbreviation identifiers.
Timing and spatial scale	Not applicable.
Data exclusions	We manually downloaded each genome and checked the completeness of the annotation files. First, all candidate genomes were used for the synteny network construction. Next, we assessed genome quality from the phylogenomic profile matrix of all synteny clusters (where rows represent species/genomes and columns are clusters). Genomes with poor completeness and contiguity -- indicated by lighter rows (for an exmple, see Figure 5 of Zhao and Schranz, 2019, rows indicated by black arrows) -- are removed from the microsynteny network.
Reproducibility	Both simulations and use of empirical data sets show our method to be accurate, consistent, and widely applicable.<br>The overall monophyly of most clades of the synteny tree of flowering plants was strongly supported, and 110 of the 122 nodes had ≥ 95% bootstrap support. Comparisons with sequence alignment-based trees and current phylogenetic classifications show that we reconstruct very accurate and robust phylogenies.
Randomization	Not applicable.
Blinding	Not applicable.

Did the study involve field work? ☐ Yes ☒ No

Reporting for specific materials, systems and methods

We require information from authors about some types of materials, experimental systems and methods used in many studies. Here, indicate whether each material, system or method listed is relevant to your study. If you are not sure if a list item applies to your research, read the appropriate section before selecting a response.

Materials & experimental systems

n/a	Involved in the study
[x]	[ ] Antibodies
[x]	[ ] Eukaryotic cell lines
[x]	[ ] Palaeontology and archaeology
[x]	[ ] Animals and other organisms
[x]	[ ] Human research participants
[x]	[ ] Clinical data
[x]	[ ] Dual use research of concern

Methods

n/a	Involved in the study
[x]	[ ] ChIP-seq
[x]	[ ] Flow cytometry
[x]	[ ] MRI-based neuroimaging
